# Development of Small-Molecule *Trypanosoma brucei N*-Myristoyltransferase Inhibitors: Discovery and Optimisation of a Novel Binding Mode

**DOI:** 10.1002/cmdc.201500301

**Published:** 2015-09-23

**Authors:** Daniel Spinks, Victoria Smith, Stephen Thompson, David A Robinson, Torsten Luksch, Alasdair Smith, Leah S Torrie, Stuart McElroy, Laste Stojanovski, Suzanne Norval, Iain T Collie, Irene Hallyburton, Bhavya Rao, Stephen Brand, Ruth Brenk, Julie A Frearson, Kevin D Read, Paul G Wyatt, Ian H Gilbert

**Affiliations:** [a] Drug Discovery Unit, College of Life Sciences, University of DundeeSir James Black Centre, Dundee, DD1 5EH (UK) E-mail: i.h.gilbert@dundee.ac.uk

**Keywords:** human African trypanosomiasis (HAT), medicinal chemistry, *N*-myristoyltransferase, structure-based drug design, *Trypanosoma brucei*

## Abstract

The enzyme *N*-myristoyltransferase (NMT) from *Trypanosoma brucei* has been validated both chemically and biologically as a potential drug target for human African trypanosomiasis. We previously reported the development of some very potent compounds based around a pyrazole sulfonamide series, derived from a high-throughput screen. Herein we describe work around thiazolidinone and benzomorpholine scaffolds that were also identified in the screen. An X-ray crystal structure of the thiazolidinone hit in *Leishmania major* NMT showed the compound bound in the previously reported active site, utilising a novel binding mode. This provides potential for further optimisation. The benzomorpholinone was also found to bind in a similar region. Using an X-ray crystallography/structure-based design approach, the benzomorpholinone series was further optimised, increasing activity against *T. brucei* NMT by >1000-fold. A series of trypanocidal compounds were identified with suitable in vitro DMPK properties, including CNS exposure for further development. Further work is required to increase selectivity over the human NMT isoform and activity against *T. brucei*.

## Introduction

Human African trypanosomiasis (HAT), or African sleeping sickness, is endemic in sub-Saharan Africa, claiming the lives of about 10 000 people every year.[1] The disease burden in this area is substantial, with approximately 60 million people at risk of infection. HAT is caused by two subspecies of the protozoan parasite *Trypanosoma brucei gambiense* and *T. brucei rhodensiense*, which are transmitted to the human host by the bite of an infected tsetse fly. If left untreated the disease is often fatal. There is a clinical need for more effective drug therapies, because current treatments are unsatisfactory due to toxicity, treatment failures, and inappropriate dosing regimens for a rural African setting.[1–4]

**Figure 1:**
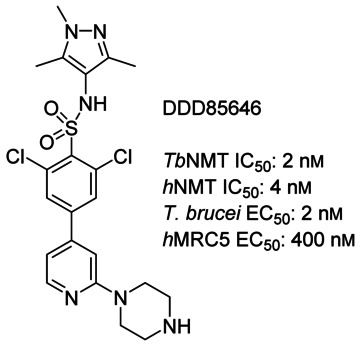
DDD85646, the previously published *T. brucei* NMT inhibitor.

The enzyme *N*-myristoyltransferase (NMT) is one of only a few genetically and chemically validated drug targets in kinetoplastids, with the NMT inhibitor DDD85646 (Figure 1) being shown to act on target and to cure the mouse model of stage-1 (non-CNS) *T. brucei* infection.[5–8] Functionally the NMT enzyme is ubiquitous and is responsible for catalysing the co-translational transfer of myristate from myristoyl-CoA to the N-terminal glycine residue of the target protein. Herein we describe the discovery and optimisation of novel *T. brucei* NMT inhibitor scaffolds identified by high-throughput screening, with appropriate physicochemical properties for oral bioavailability and CNS penetration.

## Results and Discussion

### Hit-to-lead chemistry

**Figure 2:**
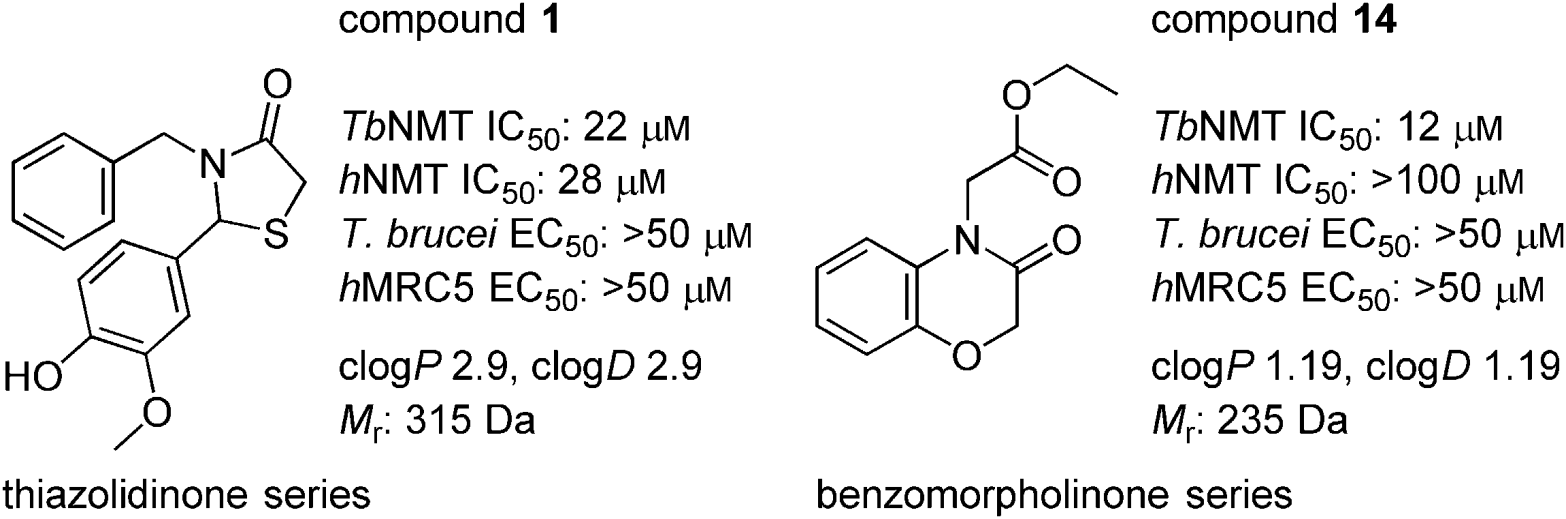
Thiazolidinone and benzomorpholinone hits.

In our initial programme to discover inhibitors of *Tb*NMT, a high-throughput screen of our diversity set was carried out. In addition to the pyrazole sulfonamide series previously reported,[8–10] several other hits were identified. These were also investigated to determine if they offer opportunities to develop selective and blood–brain barrier penetrant *Tb*NMT inhibitors. Two hits around a thiazolidinone and benzomorpholinone were identified and validated as hits (Figure 2).

**Figure 3:**
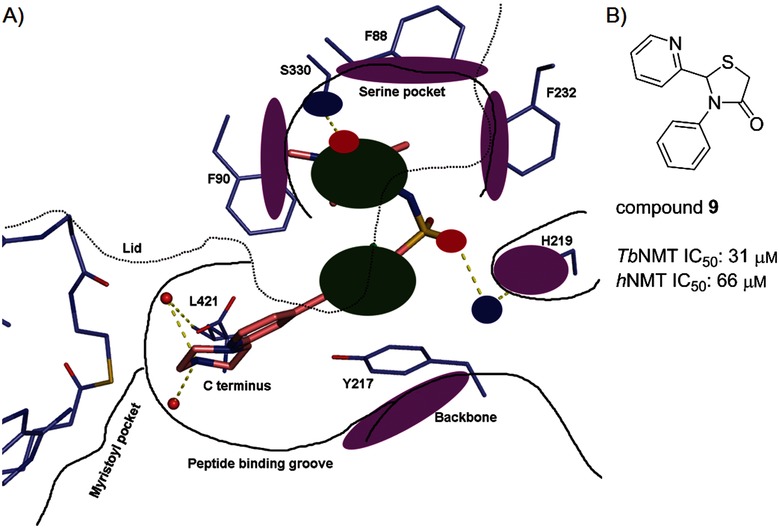
A) Structure-based pharmacophore based on the crystal structure of DDD85646 bound to *Lm*NMT. The key pharmacophoric interactions used were: 1) a hydrogen bond from the pyrazole N atom to Ser330, shown in blue; 2) a π-stacking interaction between the pyrazole and Phe90, shown in green; 3) a π-stacking interaction between the pyridine and Tyr217, shown in green; 4) a hydrogen bond between the sulfonamide O atom and His219 and Asp396. Excluded volumes are shown in purple. B) Virtual screening hit compound 9.

In parallel to a re-evaluation of the original diversity screen data, a virtual screening exercise was carried out, the basis of which was known interactions between the pyrazole sulfonamide DDD85646 and the enzyme (PDB code: 2WSA; Figure 3). Screening our in-house database of commercially available compounds using this pharmacophore led to the discovery of three different compound classes (data not shown), including examples containing the thiazoldinone scaffold (Figure 3 B).

### Thiazolidinone series

**Scheme 1:**
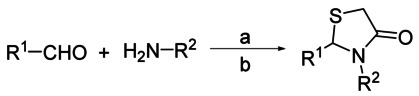
Thiazolidinone synthesis. *Reagents and conditions*: a) 1. aldehyde (2 equiv), amine (1 equiv), THF, 0 °C, 5 min; 2. thioacetic acid (3 equiv), 0 °C, 10 min; b) polymer-supported carbodiimide (1.3 equiv), 20 °C, 16 h, 33–74 %.

The initial hit expansion focused on optimising the substituents around the thiazolidinone ring. The synthetic route is shown in Scheme 1 and consisted of condensation of the aldehyde with an amine and thioacetic acid to give analogues **1**–**13** (Table 1) in yields of 33–74 %. Work was initially carried out without X-ray co-crystal structures; when this structural information was established using the *Leishmania major* enzyme, key early-stage molecules were also co-crystallised with the enzyme. As we discussed in a previous publication,[10] the *L. major* NMT shows high sequence homology to both the *T. brucei* and human NMTs. This has been an excellent system to improve the activity of inhibitors, but given the similarities and lack of high-resolution structures of the *T. brucei* enzyme, it has much less use in predicting selectivities.

**Table 1:** Initial SAR data from thiazolidinone hit expansion

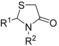							
Compd	R^1^	R^2^	IC_50_ [μm]^[a]^		EC_50_ [μm]^[a]^	LE^[b]^	Assumed
			*Tb*NMT	*h*NMT	*T. brucei*	*Tb*NMT	binding mode^[c]^
**1**	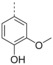	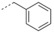	22	28	>50	0.29	T1
**2**	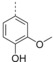	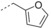	7.6	>100	>50	0.33	T1
**3**	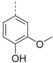	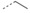	>100	>100	ND	–	–
**4**	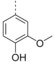	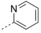	>100	>100	ND	–	–
**5**	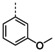	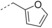	>100	>100	ND	–	–
**6**	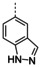	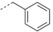	20	>100	>50	0.29	T1 (5AG5)
**7**	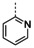	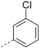	13	>50	ND	0.36	T2 (5AG4)
**8**	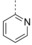	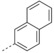	>100	>100	ND	–	T2
**9**	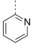	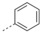	31	66	ND	0.35	T2
**10**	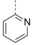	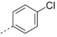	>100	>100	>50	–	–
**11**	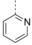	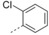	32	>100	>50	0.32	T2
**12**	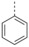	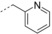	5.9	>100	>50	0.39	T1
**13**	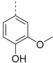	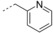	0.27	>100	14	0.42	T1 (5AG6)

**Figure 4:**
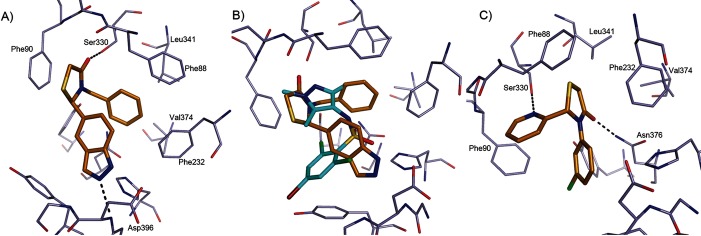
Binding mode of thiazolidinone-based ligands to *Lm*NMT. A) Compound 6 (C atoms gold) binding to *Lm*NMT (C atoms grey), adopting binding mode T1. Hydrogen bonds are shown as dashed lines, and key residues are labelled. B) Overlay of the binding mode of 6 with a pyrazole sulfonamide ligand (C atoms cyan, PDB code: 4A30).[9] C) Compound 7 (C atoms gold) binding to *Lm*NMT (C atoms grey), adopting binding mode T2. The image was prepared with PyMOL.

[a] Values shown are the mean of two or more determinations; ND=not determined. [b] Ligand efficiency (LE), determined for compounds with *T. brucei* NMT potency <50 μm, was calculated as 0.6 ln(IC_50_)/(heavy atom count).[11] [c] The assumed binding mode of each analogue is classified into either of the two specific binding modes identified by X-ray crystallography (see Figure 4); this assumption was supported by the observed SAR data and by modelling these analogues in PyMOL.

The benzyl ring at R^2^ could be replaced with a furanyl methyl group (compound **2**). However, replacement with a nonaromatic group (R^2^=ethyl, **3**) led to a complete loss of activity. Similarly, a directly attached pyridine moiety also led to a loss of activity. This loss is probably due to a clash of the pyridine moiety with the protein and suboptimal filling of the hydrophobic pocket with the methyl group. A protein–ligand structure of **6** bound to *L. major* NMT (*Lm*NMT) later confirmed this methylene-aromatic group occupies a hydrophobic pocket in the active site (see binding mode T1, Figure 4 A,B).

We were keen to replace the 2-methoxyphenol group, due to the potential for de-methylation and oxidation to a quinone. Removal of the phenol group from **2**, as in compound **5**, resulted in loss of potency, indicating the importance of this group for binding. The phenol hydrogen bond donor of compounds **1** and **2** could be replaced by the indazole isostere, as in compound **6**, without loss of potency. The complex of **6** bound to *Lm*NMT revealed binding mode T1 of the thiazolidinone series, (Figure 4). The thiazolidinone ligand occupies a similar area of the peptide binding pocket of NMT as the pyrazole sulfonamide ligands[8] (Figure 4 B). In binding mode T1, the thiazolidinone core of **6** packs against the side chain of Phe90, and the carbonyl group forms a key hydrogen bonding interaction with the side chain of Ser330. The benzyl moiety of **6** forms π-stacking interactions with the side chains of Phe88 and Phe232 (parallel and edge–face, respectively), with the pocket enclosed by the side chains of Leu341 and Val374. The indazole N2 lone pair forms a hydrogen bond to the backbone amide of Asp396, a key interaction identified in the pyrazole sulfonamide series. Despite the ligand being prepared without chiral resolution, only the *R* enantiomer was observed bound in the crystal structure. Compounds **1** and **2** were assumed to have a similar binding mode.

Simultaneous replacement of the R^1^ 3-phenol-4-methoxy groups of **1** with a 2-pyridyl unit, and truncation of the R^2^ benzyl group to a directly linked phenyl, resulted in compound **7** and an unexpected inversion of the binding mode from that observed for **6**, giving rise to binding mode T2 (Figure 4 C). Compounds that adopted binding mode T2 show the R^1^ 2-pyridyl subunit forming a hydrogen bonding interaction with the side chain of Ser330, and the thiazolidinone carbonyl group forming a hydrogen bonding interaction with the side chain of Asn376. The X-ray crystal structure also revealed the R^2^ substituent to be located in the hydrophobic peptide binding groove, lying in a similar plane to the aryl group in the pyrazole sulfonamide series (Figure 4). In binding mode T2, and in contrast to **6**, the *S* enantiomer was bound in the active site. It is unclear why compound **7** displayed selectivity for *Tb*NMT over *h*NMT (Table 1). However, this observed selectivity was lost upon removal of the (R^2^-phenyl)-3-chloro substituent, seen with **9**, although this is partly due to the overall diminished potency observed for this compound. Substitution at the *ortho* (**11**) or *meta* (**12**) positions of the R^2^ phenyl group appeared to be preferred over *para* substitution (**10**) which may be due to a clash of this substituent with the side chain of Tyr217.

**Figure 5:**
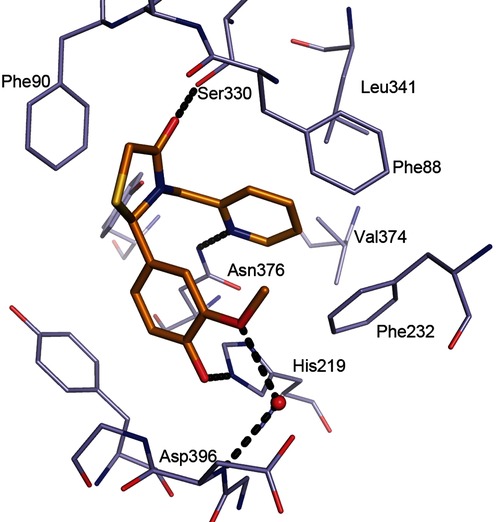
Binding of 13 to *Lm*NMT. Hydrogen bonds are shown as dashed lines, and key residues are labelled.

During our exploration of the structure–activity relationship (SAR) around the thiazolidinone scaffold, the 2-pyridylmethylene subunit of **12** was identified as the most ligand efficient R^2^ substituent (LE=0.39; Table 1). Modelling of **12** into the binding sites of **6** and **7** could explain the efficiency of binding. Assuming **12** adopted binding mode T1, there was no clear ligand–protein interaction with the His219 residue; however, we postulated a hydrogen bonding interaction between the ligand and residue Asn376, with the R^2^ 2-pyridyl nitrogen atom as the hydrogen bond acceptor. Compound **13** was synthesised; it is a hybrid of compounds **12** and **1**, with the addition of 4-hydro-3-methoxy to create an additional hydrogen bond with His219, seeking to afford a significant improvement in potency. X-ray crystallography confirmed **13** as achieving this interaction and a significant improvement in potency (20-fold, IC_50_: 0.27 μm) and a slight improvement in ligand efficiency to 0.42 (Figure 5 and Table 1).

The thiazolidinone series, in particular compound **13**, presents a good starting point for further optimisation. It should be possible to gain additional affinity by accessing an additional local binding interaction or filling a local hydrophobic pocket by using a structure-based design approach. Replacement of the phenol subunit with a bioisostere has proved achievable; this substituent could be further optimised.[12] Most compounds were not sufficiently potent against the enzyme to give significant activity in the parasite assays. Compound **13**, the most potent compound, showed a 50-fold decrease in activity in going from enzyme to cell. However, further work is required to derive compounds with greater enzyme and parasite activity.

### Benzomorpholinone series

**Figure 6:**
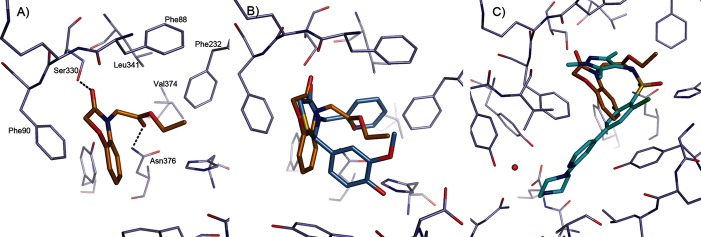
Binding mode of benzomorpholinone ligands. A) Binding mode of 14 (C atoms gold) to *Lm*NMT. B) Similarity in core binding of the benzomorpholinone of 14 (C atoms gold) and the thiazolidinone of 13 (C atoms blue). C) Binding mode of 14 (C atoms gold) compared with the pyrazole sulfonamide ligand DDD85646 (C atoms cyan, PDB code: 2WSA).[8] The image was prepared using PyMOL.

The hit compound **14** (Figure 2) has a low molecular weight (*M*_r_=235 Da), good ligand efficiency (LE=0.42), and provided an excellent starting point for further optimisation (Table 2 and Figure 6). The initial optimisation focus for the benzomorpholinone series was to identify replacements for the potentially hydrolysable ester functional group of **14**.

**Table 2:** Initial SAR data from benzomorpholinone hit expansion

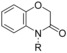				
Compd	R	IC_50_ [μm]^[a]^		LE^[b]^
		*Tb*NMT	*h*NMT	*Tb*NMT
**14**	CH_2_COOEt	12	>100	0.42
**15**	CH(CH_3_)COOEt	>100	>100	–
**16**	CH_2_CH_2_COOEt	13	>100	0.38
**17**	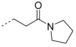	11	>100	0.34
**18**	CH_2_CH_2_CON(CH_3_)_2_	50	>100	–
**19**	CH_2_CON(CH_3_)_2_	>100	>100	–
**20**	CH_2_CONHCH_3_	>100	>100	–
**21**	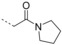	>100	ND	–
**22**	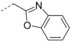	24	>100	0.31
**23**	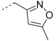	2.9	>100	0.43
**24**	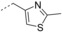	44	>100	0.33
**25**	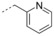	22	>100	0.38
**26**	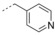	>100	>100	–
**27**	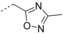	>100	>100	–
**28**	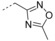	7.0	>100	0.40
**29**	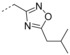	18	>100	0.32
**30**	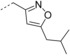	9	30	0.34
**31**	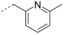	41	69	0.31
**32**	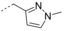	80	>100	–

[a] Values shown are the mean of two or more determinations; ND=not determined. [b] Ligand efficiency (LE), determined for compounds with *T. brucei* NMT potency <50 μm, was calculated as 0.6 ln(IC_50_)/(heavy atom count).[11]

#### Synthetic route to the benzomorpholinone series

**Scheme 2:**
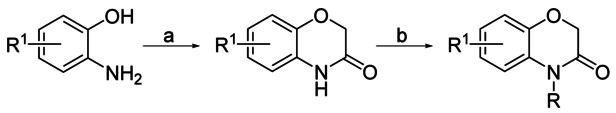
Benzomorpholinone synthesis. *Reagents and conditions*: a) 2-chloroacetyl chloride (1 equiv), 2-aminophenol (1 equiv), K_2_CO_3_ (1 equiv), DMF, 80 °C, 3 h; b) K_2_CO_3_ (1.4 equiv), DMF, 70 °C, 18 h; overall yield: 30–70 % (two steps).

Chloroacetylchloride was reacted with the appropriately substituted 2-aminophenol to give the NH benzomorpholinone intermediate (Scheme 2). This was then alkylated with potassium carbonate as the base, with heating in DMF. Workup and purification yielded compounds **14**–**38**.

Compound **14** was successfully co-crystallised with *Lm*NMT. The complex shows the ligand bound in a an orientation similar to that of the thiazolidinone ligand **13**, creating a very similar interaction pattern (Figure 6). The benzomorpholinone packs against the side chain of Phe90 with the carbonyl forming a hydrogen bond to the side chain of Ser330 (Figure 6 A). The carbonyl group of the ester moiety forms a hydrogen bond interaction with the side chain of Asn376, occupying a similar position to the pyridyl group of compound **13** (Figure 6 B).

On the fragment level, a one- (C1, **14**) or two- (C2, **16**) carbon linker to the ester was equally well tolerated, with *Tb*NMT IC_50_ potencies around the 10 μm level. The labile ester functional group was replaced by a pyrrolidine amide (compound **17**) without loss of potency, although the dimethylamino amide (compound **18**) showed a fivefold decrease in potency. It was also found that C1-linked amides **19**, **20**, and **21** lost all potency. Although the reason for these significant potency losses is unclear, the decrease in lipophilicity for these particular compounds may be partly responsible for the lower potencies observed.

C1-linked heterocyclic isosteres for the ester compound **14** were tolerated, although there was a requirement for a heteroatom in the α position, presumably to act as a hydrogen bond acceptor (HBA) with the side chain of Asn376. The vector and relative hydrogen bond potential of the lone pair on the HBA atom, coupled with the orientation of the alkyl substituent appeared critical drivers for potency. The heterocyclic isostere with the highest potency was the methyl-isoxazole **23** (*Tb*NMT IC_50_: 2.9 μm). This compound displayed a fourfold improvement in *Tb*NMT potency over **14**. The thiazole (**24**), pyrazole (**32**), and oxadiazole (**27**) heterocyclic analogues lost significant potency relative to compound **23**. In the case of **24** and **32**, we hypothesised from our binding models that the methyl group is suboptimally positioned, and for oxadiazole **27** the oxygen is a relatively weak HBA moiety. This explanation was further supported by the recovery in potency observed for the isomeric oxadiazole **28** (*Tb*NMT IC_50_: 7.0 μm). In this oxadiazole isomer **28**, the nitrogen atom presumably acts as the HBA motif, and is predicted to have a similar HBA potential as the isoxazole nitrogen atom in **23**.[13,14] There was no potency gain observed upon adding further lipophilic bulk to the methyl group in the β position of the heterocycle, for example homologation to isobutyl analogue in **29** or **30**. Furthermore, for both compounds this substitution decreased the LE significantly.

The 2-pyridyl analogue **25** retained the potency of the ester **14**, but was found to be nearly 10-fold less potent than **23**. This was not predicted by the HBA potential. The reason for the loss in potency might be that the methyl group contributes favourably to binding, or alternatively this heterocycle does not achieve an optimised hydrogen bonding interaction with the protein due to an unfavourable HBA orientation. Further potency (relative to **23**) was lost when the methyl group was added at a suboptimal position (α to the heteroatom) as in compound **31**, and all potency was lost with the 4-pyridyl isomer **26**, presumably due to loss of the hydrogen bond with Asn376.

#### Benzomorpholinone hit-to-lead strategy

Compound **23** showed similar potency and LE as those of the initial hit **14**, and lacks the metabolically and chemically labile ester functional group. This scaffold was developed further in a hit-to-lead campaign to investigate other substitution vectors using a rational structure-based design approach based on X-ray crystallography data. This approach used the binding mode overlay of the pyrazole sulfonamide inhibitor DDD85646 with **14** (Figure 6). From our previous work on the pyrazole sulfonamide series, appending a basic moiety to reach the C-terminal carboxylic acid gave an approximate 1000-fold increase in potency.[8–10] Using this hypothesis, cross-over compounds were designed to target nanomolar efficacy in the parasite growth assay, good pharmacokinetic parameters, and selectivity for parasite efficacy (relative to *h*NMT enzyme inhibition) to identify compounds suitable for evaluation in an animal model of HAT. The selectivity ratio of *T. brucei* cell versus *h*NMT enzyme potency was used as a prediction for the in vivo therapeutic window in the pyrazole sulfonamide series, and formed the basis of ranking and selecting a back-up series for lead optimisation (Table 3).[10]

**Table 3:** Benzomorpholinone series hit to lead optimisation

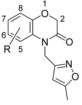							
Compd	R	IC_50_ [μm]^[a]^		EC_50_ [μm]^[a]^		Selectivity	LE^[b]^
		*Tb*NMT	*h*NMT	*T. brucei*	MRC5	ratio	*Tb*NMT
**33**	H	2.9	>100	ND	ND	–	0.43
**34**	2-CH_3_	>100	>100	ND	ND	–	–
**35**	6-Chloro	>100	>100	ND	ND	–	–
**36**	7-Chloro	7.1	>100	ND	ND	–	0.37
**37**	7-Bromo	6.4	>100	ND	ND	–	0.37
**38**	8-Bromo	11	>100	ND	ND	–	0.37
**39**	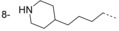	0.008	0.7	0.41	>50	2	0.40
**40**	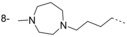	<0.002	1.0	0.08	24	13	>0.38
**41**	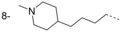	<0.002	0.9	0.19	19	5	>0.40
**42**	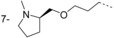	0.009	24	0.35	>50	69	0.38
**43**	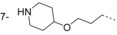	<0.002	0.9	0.15	22	6	>0.40
**44**	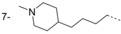	<0.002	0.04	0.007	10	6	>0.40

[a] Values shown are the mean of two or more determinations; ND=not determined. [b] Ligand efficiency (LE), determined for compounds with *T. brucei* NMT potency <50 μm, was calculated as 0.6 ln(IC_50_)/(heavy atom count) for *Tb*NMT.[11] [c] Selectivity ratio: *h*NMT (EC_50_)/*T. brucei* cell (EC_50_).

#### Hit-to-lead discussion

Investigation of simple halogen and methyl group substitution around the core benzomorpholinone scaffold indicated substitution with a halogen (Cl or Br), as in **36** and **37**, is tolerated at the 7-position (Table 3). The 8-bromo analogue **38** showed similar potency to 7-bromo compound **37**. This implies that the 7- or 8-positions are suitable vectors for extension of **23** toward the C terminus, in agreement with the X-ray crystal structure (Figure 6).

**Figure 7:**
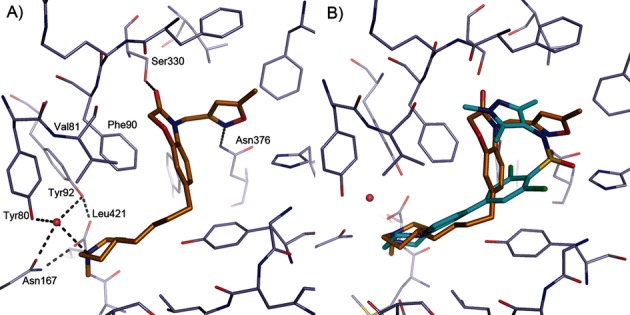
Crystallographically determined binding mode of 44 bound to *Lm*NMT. A) Orientation of 44 (C atoms gold) bound to *Lm*NMT. Hydrogen bonds are shown as dashed lines. B) Comparison of 44 with the pyrazole sulfonamide compound DDD85646 (C atoms cyan, PDB code: 2WSA). The image was prepared using PyMOL.

Extended analogues, postulated to achieve the interaction with the C-terminal residue in the active site, were designed and synthesised. Data are listed in Table 3. These analogues were designed to interact directly, or through a water-mediated hydrogen bond, with the C-terminal carboxylate group to obtain potency gains as observed for the previously reported pyrazole sulfonamide series.[8,9] Crystal structure analysis of **44** in complex with *Lm*NMT confirmed that this interaction was achieved (Figure 7). The benzomorpholinone core of **44** retained the binding mode of the original hit **14**, with the methyl isoxazole packing against Phe232 and the nitrogen lone pair forming a hydrogen bond to Asn376. The substituent at the 7-position of the benzomorpholinone extended down the peptide binding groove presenting the methyl-piperidine group toward the C-terminal carboxylate. The piperidine nitrogen did not interact directly with the protein chain, but hydrogen bonded to a highly coordinated water molecule, an interaction observed in previous series of NMT inhibitors.[8,9] The potencies of the extended analogues **39**–**44** all indicate this interaction has been achieved, with all six analogues showing >100-fold improvement in potency at *Tb*NMT.

Notably, potencies of <0.002 μm were at the limit of the enzyme assay′s sensitivity; compounds at this end of the potency range are probably sub-nanomolar inhibitors. In previous works[8–10] we reported that there is a good correlation between activity against the parasite and inhibition of *Tb*NMT, so we reported a selectivity measure using the EC_50_ value against the parasite in predicting selectivity.[10] Relative selectivities calculated as (*h*NMT IC_50_)/(*T. brucei* cell assay EC_50_) were used to compare compounds.[10] Whilst it is unknown if inhibition of mammalian NMT is dose limiting or whether the compounds have off-target effects, this “selectivity” value turned out to be better at predicting the “therapeutic ratio” in mouse models of infection than a simple ratio of cellular activities. For example, **44** was found to be >10-fold more potent in the parasite growth assay than the other five extended compounds. Unfortunately, the *h*NMT potency of **44** was correspondingly increased as well (*h*NMT IC_50_: 0.04 μm), so although the cellular selectivity (MRC5 cell/*T. brucei* cell) was promising at 1400-fold, the more informative *h*NMT IC_50_/*T. brucei* cell assay EC_50_ (in vivo predictive) selectivity index was only sixfold. Compound **40** had the highest *h*NMT IC_50_/*T. brucei* cell assay EC_50_ selectivity index of 13-fold, but unfortunately this compound had a poorer *T. brucei* cellular efficacy (EC_50_: 80 nm) and was not sufficiently potent to progress into an in vivo efficacy study.

#### Pharmacokinetic and physicochemical properties of the benzomorpholinone series

The lead compounds showed a good balance of physicochemical properties (Table 4), which were within acceptable lead optimisation limits. Lipophilicity was clearly higher for the *N*-methyl analogues, although the clog*D* values were still acceptable. The mouse hepatic microsomal intrinsic clearance was high for *N*-methyl analogues, most probably driven by N-demethylation to the secondary amines. The secondary amines (e.g., **39**) were inherently more metabolically stable and still showed enzyme and *T. brucei* cellular activity in the nanomolar range (Table 3).

**Table 4:** In vitro pharmacokinetic and physicochemical properties

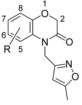						
Compd	R	*M*_r_ [Da]	clog*P*/clog*D*^[a]^	PSA [Å^2^]^[a]^	*CL*_int_ (m)^[b]^	PPB [%]^[b]^
**39**	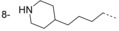	383	3.8/1.2	68	2.0	ND
**40**	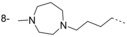	412	3.1/1.0	62	ND	ND
**41**	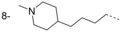	397	4.0/1.7	59	10	82
**42**	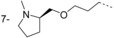	399	2.7/1.0	68	7.0	ND
**43**	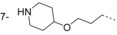	385	2.8/0.6	77	ND	ND
**44**	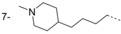	397	4.0/1.7	59	12	94

[a] Polar surface area, calculated partition (clog*P*) and distribution (clog*D*) coefficients calculated using *StarDrop* from Optibrium. [b] *CL*_int_ (m) [mL min^−1^ (g liver)^−1^] and plasma protein binding (PPB) were determined using the protocol described in the Experimental Section; ND=not determined.

Interestingly, we observed that the vector of substitution from the benzomorpholinone core had a substantial effect on the plasma protein binding (PPB) of these compounds and their ability to cross the blood–brain barrier in in vivo studies, where the 8-substituted compound had a much higher brain-to-blood ratio (B/B=27) than the 7-substituted compound (B/B=0.12). The latter property is essential for progression into lead optimisation and for candidates suitable for clinical trials, as the parasite has already invaded the CNS before most patients present for treatment.

## Conclusions

Compounds around two different cores, thiazolidinones and benzomorpholinones, were validated as hits for *Tb*NMT. The benzomorpholinones originated from HTS, and the thiazolidinones were discovered by HTS and virtual screening in parallel. Interestingly, the thiazolidinone compounds displayed two distinct binding modes with different enantiomers bound (Figure 4). One of the binding modes was very similar to that adopted by the benzomorpholinones (Figures 6 and 7). All binding modes of the described compounds differ from the binding modes of the previously described pyrazole sulfonamide compounds (Figures 4 B and 7 B), or a number of structures of *Leishmania* NMT with various ligands that were published subsequent to the work reported herein.[15,16] This enabled us to explore new vectors and interactions when attempting to optimise the affinities of the hit compounds.

The thiazolidinone hit **1**, which displayed relatively weak *Tb*NMT potency with no selectivity over *h*NMT, was expanded into a hit series of compounds using a one-pot, two-step reaction. The indazole isostere **6** for the methoxyphenol was quickly identified as being equipotent and more selective (displaying no measurable inhibition of *h*NMT). The discovery of a binding mode switch for the thiazolidinone in compound **7** and the highly ligand efficient lead **13** provides a potential starting point for lead optimisation chemistry, although cellular efficacy was a potential issue for this series (best in class *T. brucei* EC_50_: 6.3 μm). Further optimisation of **13** should be possible with a structure-based design approach to target additional interactions in the binding site. Alternatively, following on from SAR observations with the pyrazole sulfonamide and benzomorpholinone series, a hydrogen bonding/salt bridge ligand–protein interaction with the C-terminal carboxylate group could be targeted. This should lead to nanomolar *Tb*NMT potencies (as previously observed), which should afford potent parasite cell activities at *T. brucei*.[8,9] A suitable group could be designed, with correct vector and linker to achieve this interaction, as seen in the benzomorpholinone series hit-to-lead strategy. However, this strategy is not without risk, because data appear to suggest that targeting the C-terminal residue for a ligand–protein interaction may also result in correspondingly potent *h*NMT inhibition, as observed with the benzomorpholinone series (Table 3). This could lead to potential issues with selectivity and toxicity (through mammalian NMT inhibition). The thiazolidinone scaffold also offers the opportunity to explore previously unexplored vectors, with the possibility of identifying new protein–ligand interactions which could deliver selective potency at *Tb*NMT (over *h*NMT).

On the benzomorpholinone series, we started with the low-molecular-weight, ligand-efficient hit compound **14**. We identified several bioisosteric replacements of the chemically labile ester functionality, two of which additionally displayed improved *Tb*NMT potency and good ligand efficiency. A structure-based drug design approach was instigated, using **23** as the parent template for substitution. Vectors for attaining substitution toward the protein's C terminus were identified by X-ray crystallography and from initial SAR investigations around the core scaffold. Six compounds, **39**–**44**, were described which appear to have achieved this additional ligand–protein interaction, and which showed the expected increase in potency at *Tb*NMT. Within this series we developed *Tb*NMT enzyme potency from the 10 μm level to <0.002 μm. The enzyme and cellular potencies correlated directly, indicating an on-target effect of killing the parasites in culture. Due to high structural homology between the human and *T. brucei* NMT enzyme orthologues, improvements in potency toward the sub-10 nm levels at *Tb*NMT were often accompanied by a corresponding improvement in *h*NMT inhibition (Table 3). Compound **42** gave some indication that *Tb*NMT/*h*NMT selectivity may be achievable within this series, although **42** itself had insufficient cellular potency to progress further and into in vivo efficacy studies.

In compounds from these two series there was a 30- to 50-fold decrease in activity going from *Tb*NMT enzyme to *T. brucei* cell efficacy, which further narrowed selectivity over mammalian NMT inhibition; this may have the potential to drive toxicity. Compounds from the benzomorpholinone series afforded potent antiparasitic cell activities (*T. brucei* EC_50_: 0.007 μm for **44**); they display promising in vitro DMPK profiles, showing this series has the potential to be optimised further to deliver orally active antiparasitic compounds. The 8-substituted benzomorpholinone series in particular shows good potential for stage-2 in vivo efficacy, although improvements in selectivity would be needed to extend the therapeutic window and to allow higher dose levels in order to maximise the chances of curing stage-2 infected mice. It is unclear at this time how to rationally achieve improved selectivity to the required levels, although this could be possible by using currently undiscovered selectivity pockets and protein secondary structure differences between *T. brucei* and human NMT.

Starting from the singleton HTS hits **1** and **14**, and the virtual screening hit **9**, we have described the development of two potent *Tb*NMT enzyme inhibitor series, which provide good starting points for drug discovery programmes for the treatment of stage-1 and stage-2 HAT. These provide novel scaffolds and give opportunities for exploring new vectors.

## Experimental Section

### Chemistry

Chemicals and solvents were purchased from Aldrich Chemical Co., Fluka, ABCR, VWR, Acros, Fisher Chemicals, and Alfa Aesar, and were used as received unless otherwise stated. Air- and moisture-sensitive reactions were carried out under an inert atmosphere of argon in oven-dried glassware. Analytical thin-layer chromatography (TLC) was performed on pre-coated TLC plates (layer 0.20 mm silica gel 60 with fluorescent indicator UV254, Merck). Developed plates were air dried and analyzed under a UV lamp (*λ* 254/365 nm). Flash column chromatography was performed using prepacked silica gel cartridges (230–400 mesh, 40–63 mm, SiliCycle) (unless otherwise stated) using a Teledyne ISCO Combiflash Companion or Combiflash Retrieve. ^1^H and ^13^C NMR spectra were recorded on a Bruker Avance II 500 spectrometer (^1^H at 500.1 MHz, ^13^C at 125.8 MHz), or a Bruker DPX300 spectrometer (^1^H at 300.1 MHz). Chemical shifts (*δ*) are expressed in ppm recorded using the residual solvent as internal reference in all cases. Signal splitting patterns are described as singlet (s), doublet (d), triplet (t), quartet (q), pentet (p), multiplet (m), broad (br), or a combination thereof. Coupling constants (*J*) are quoted to the nearest 0.1 Hz. LC–MS analyses were performed with either an Agilent HPLC 1100 series instrument connected to a Bruker Daltonics MicrOTOF or an Agilent Technologies 1200 series HPLC connected to an Agilent Technologies 6130 quadrupole spectrometer, where both instruments were connected to an Agilent diode array detector. LC–MS chromatographic separations were conducted with a Waters Xbridge C_18_ column, 50 mm×2.1 mm, 3.5 mm particle size; mobile phase: H_2_O/MeCN+0.1 % HCOOH, or H_2_O/MeCN+0.1 % NH_3_; linear gradient from 80:20 to 5:95 over 3.5 min and then held for 1.5 min; flow rate: 0.5 mL min^−1^. All tested compounds had a measured purity of 95 % (by TLC and UV) as determined by this analytical LC–MS system. High-resolution electrospray MS measurements were performed on a Bruker Daltonics MicrOTOF mass spectrometer. Microwave-assisted chemistry was performed using a Biotage initiator microwave synthesiser.

**General procedure for the synthesis of thiazolidinones**: To a cooled solution of aldehyde (2 mmol) in THF (5 mL) at 0 °C, was added amine (1 mmol) and the reaction was stirred for 5 min. Thioacetic acid (3 mmol) was then added, and stirring continued for 10 min. Polymer-supported carbodiimide (1.33 mmol, 1.33 mmol g^−1^ loading) was then added, the reaction warmed to RT, and stirring continued for 16 h. The reaction was filtered, the resin washed (THF, MeOH) and the filtrate concentrated in vacuo. The resultant crude residue was taken up in CH_2_Cl_2_, washed (10 % citric acid (aq), water, saturated sodium bicarbonate (aq), brine), dried (MgSO_4_) and concentrated in vacuo. The resultant crude residue was purified by column chromatography (0–100 % EtOAc/hexane) to give the title compound.

**General procedure for the synthesis of benzomorpholinones**: Appropriate 2-chloroacetyl chloride (21.3 mmol) was added dropwise to a mixture of appropriate 2-aminophenol (21.3 mmol) and K_2_CO_3_ (21.3 mmol) in DMF (40 mL). The reaction mixture was heated at 80 °C for 3 h. The solvent was then removed in vacuo, the residue was partitioned with CH_2_Cl_2_ and water, and the organics concentrated. The solid obtained was triturated and filtered with Et_2_O to give substituted-2*H*-benzo[*b*][1,4]oxazin-3(4*H*)-one.

Appropriately substituted-2*H*-benzo[*b*][1,4]oxazin-3(4*H*)-one (0.67 mmol) was taken into DMF (5 mL). Alkyl halide (0.74 mmol) and anhydrous K_2_CO_3_ (1.01 mmol) were added and stirred at 60 °C overnight. The reaction mixture was concentrated in vacuo, the residue partitioned between CH_2_Cl_2_ and saturated aqueous NaHCO_3_, the organics dried (MgSO_4_) and concentrated to dryness. Purification by column chromatography (0–5 % MeOH/CH_2_Cl_2_) gave the title compounds as solids.

#### Thiazolidinone series compounds

**3-Benzyl-2-(4-hydroxy-3-methoxyphenyl)thiazolidin-4-one (1)**: Prepared using benzylamine (0.11 mL, 1 mmol) and vanillin (304 mg, 2 mmol). Compound **1** was obtained as an off-white solid (233 mg, 74 %): ^1^H NMR (500 MHz, [D_6_]DMSO): *δ*=9.23 (s, 1 H), 7.33–7.24 (m, 3 H), 7.11–7.07 (m, 2 H), 6.81 (d, *J*=2.0 Hz, 1 H), 6.77–6.70 (m, 2 H), 5.48 (d, *J*=1.6 Hz, 1 H), 4.76 (d, *J*=15.3 Hz, 1 H), 3.93 (dd, *J*=1.6 and 15.5 Hz, 1 H), 3.78–3.67 ppm (m, 5 H); LC–MS: *m*/*z*=316 [*M*+H].

**3-(Furan-2-ylmethyl)-2-(4-hydroxy-3-methoxyphenyl)thiazolidin-4-one (2)**: Prepared with furfurylamine (0.088 mL, 1 mmol) and vanillin (304 mg, 2 mmol). Compound **2** was obtained as an off-white solid (202 mg, 66 %): ^1^H NMR (500 MHz, [D_6_]DMSO): *δ*=9.23 (s, 1 H), 7.58 (dd, *J*=0.9 and 1.8 Hz, 1 H), 6.85 (d, *J*=1.7 Hz, 1 H), 6.75–6.71 (m, 2 H), 6.38 (dd, *J*=1.8 and 3.5 Hz, 1 H), 6.15 (dd, *J*=0.8 and 3.3 Hz, 1 H), 5.51 (d, *J*=1.6 Hz, 1 H), 4.74 (d, *J*=15.3 Hz, 1 H), 3.93 (dd, *J*=1.6 and 15.5 Hz, 1 H), 3.77–3.67 ppm (m, 5 H); LC–MS: *m*/*z*=306 [*M*+H].

**3-Ethyl-2-(4-hydroxy-3-methoxyphenyl)thiazolidin-4-one (3)**: Prepared using 2 m ethylamine in THF (0.5 mL, 1 mmol) and vanillin (304 mg, 2 mmol). Compound **3** was obtained as an off-white solid (155 mg, 61 %): ^1^H NMR (500 MHz, [D_6_]DMSO): *δ*=9.22 (s, 1 H), 6.94 (d, *J*=2.0 Hz, 1 H), 6.81 (dd, *J*=2.1 and 8.2 Hz, 1 H), 6.77 (d, *J*=8.0 Hz, 1 H), 5.74 (d, *J*=1.7 Hz, 1 H), 3.82 (dd, *J*=1.6 and 15.5 Hz, 1 H), 3.77 (s, 3 H), 3.64 (d, *J*=15.2 Hz, 1 H), 3.44 (m, 1 H), 2.67 (m, 1 H), 0.91 ppm (t, *J*=7.2 Hz, 3 H); LC–MS: *m*/*z*=254 [*M*+H].

**2-(4-Hydroxy-3-methoxyphenyl)-3-(pyridin-2-yl)thiazolidin-4-one (4)**: Prepared using 2-aminopyridine (94 mg, 1 mmol) and vanillin (304 mg, 2 mmol). Compound **4** was obtained as an off-white solid (200 mg, 66 %): ^1^H NMR (500 MHz, [D_6_]DMSO): *δ*=9.04 (s, 1 H), 8.31 (m, 1 H), 7.91–7.86 (m, 1 H), 7.85–7.81 (m, 1 H), 7.18–7.13 (m, 1 H), 6.92 (d, *J*=2.0 Hz, 1 H), 6.75 (s, 1 H), 6.70 (dd, *J*=2.0 and 8.3 Hz, 1 H), 6.62 (d, *J*=8.2 Hz, 1 H), 4.08 (dd, *J*=1.1 and 16.0 Hz, 1 H), 3.87 (d, *J*=16.0 Hz, 1 H), 3.71 ppm (s, 3 H); LC–MS: *m*/*z*=303 [*M*+H].

**3-(Furan-2-ylmethyl)-2-(3-methoxyphenyl)thiazolidin-4-one (5)**: Prepared using Furfurylamine (0.088 mL, 1 mmol) and 3-methoxybenzaldehyde (272 mg, 2 mmol). Compound **5** was obtained as a clear colourless oil (93 mg, 33 %): ^1^H NMR (500 MHz, [D_6_]DMSO): *δ*=7.58 (dd, *J*=0.9 and 1.8 Hz, 1 H), 7.38 (t, *J*=7.8 Hz, 1 H), 6.95–6.86 (m, 3 H), 6.38 (dd, *J*=1.8 and 3.5 Hz, 1 H), 6.17 (dd, *J*=0.8 and 3.3 Hz, 1 H), 5.58 (d, *J*=1.6 Hz, 1 H), 4.80 (s, 0.5 H), 4.77 (s, 0.5 H), 3.94 (d, *J*=1.6 Hz, 0.5 H), 3.91 (d, *J*=1.6 Hz, 0.5 H), 3.70–3.60 ppm (m, 5 H); LC–MS: *m*/*z*=290 [*M*+H].

**3-Benzyl-2-(1*H*-indazol-6-yl)thiazolidin-4-one (6)**: Prepared using benzylamine (0.11 mL, 1 mmol) and indazole-5-carbaldehyde (292 mg, 2 mmol). Compound **6** was obtained as an off-white solid (155 mg, 50 %): ^1^H NMR (500 MHz, [D_6_]DMSO): *δ*=13.15 (s, 1 H), 8.07 (m, 1 H), 7.68 (m, 1 H), 7.35–7.25 (m, 4 H), 7.10 (m, 1 H), 7.33 (m, 1 H), 5.68 (d, *J*=1.4 Hz, 1 H), 4.84 (d, *J*=15.4 Hz, 1 H), 4.01 (dd, *J*=1.5 and 15.7 Hz, 1 H), 3.87 (d, *J*=15.8 Hz, 1 H), 3.71 ppm (s, 3 H); LC–MS: *m*/*z*=303 [*M*+H].

**3-(2-Chlorophenyl)-2-(pyridin-2-yl)thiazolidin-4-one (11)**: Prepared using 3-chloroaniline (382 mg, 4 mmol) and pyridine-2-carbaldehyde (214 mg, 2 mmol). Compound **11** was obtained as an off-white solid (290 mg, 50 %): ^1^H NMR (500 MHz, [D_6_]DMSO): *δ*=8.58 (br s, 1 H), 7.78–7.82 (m, 1 H), 7.53–7.59 (m, 2 H), 7.31–7.38 (m, 2 H), 7.25 (m, 1 H), 7.05 (br s, 1 H), 6.14 (br s, 1 H), 4.09 (dd, *J*=15.6, 1.4 Hz, 1 H), 3.82 ppm (d, *J*=15.5 Hz, 1 H); LC–MS: *m*/*z*=291 [*M*+H].

**3-(4-Chlorophenyl)-2-(pyridin-2-yl)thiazolidin-4-one (10)**: Prepared using 4-chloroaniline (382 mg, 4 mmol) and pyridine-2-carbaldehyde (214 mg, 2 mmol). Compound **10** was obtained as a beige solid (250 mg, 43 %): ^1^H NMR (500 MHz, [D_6_]DMSO): *δ*=8.49 (m, 1 H), 7.58 (m, 1 H), 7.13–7.20 (m, 6 H), 6.02 (d, *J*=1.3 Hz, 1 H), 4.01 (dd, *J*=16, 1.3 Hz, 1 H), 3.73 ppm (d, *J*=16 Hz, 1 H); LC–MS: *m*/*z*=291 [*M*+H].

**2-Phenyl-3-(pyridin-2-ylmethyl)thiazolidin-4-one (12)**: A solution of 2-pyridylmethylamine (412 μL, 4 mmol) and benzaldehyde (305 μL, 3 mmol) in EtOH (12 mL) was heated in a microwave at 100 °C for 10 min. Thioacetic acid (2 mmol) was added to the reaction, which was heated for a further 10 min at 100 °C. Zinc chloride (2 mmol) was then added and the reaction mixture was heated at high absorbance setting (in microwave) at 100 °C for 15 min, before being cooled and transferred into a separating funnel. EtOAc (30 mL) and 1 n sodium hydroxide (30 mL) was added to the separating funnel, which was then shaken. The organic layer was collected and washed with brine (2×30 mL), then dried with MgSO_4_. The solvent was removed in vacuo and the crude mixture was purified by column chromatography (0–6 % MeOH/CH_2_Cl_2_) to yield compound **12** as an off-white solid (216 mg, 40 %): ^1^H NMR (500 MHz, [D_6_]DMSO): *δ*=8.5 (m, 1 H), 7.76 (m, 1 H), 7.32–7.40 (m, 5 H), 7.29 (m, 1 H), 7.20 (d *J*=8 Hz, 1 H), 5.79 (d, *J*=1.7 Hz, 1 H), 4.87 (d, *J*=16, 1 H), 3.97 (dd, *J*=15.4, 1.8 Hz, 1 H), 3.76–3.82 ppm (m, 2 H); LC–MS: *m*/*z*=271 [*M*+H].

**2-(4-Hydroxy-3-methoxyphenyl)-3-(pyridin-2-ylmethyl)thiazolidin-4-one (13)**: Prepared as for **12**, using 2-pyridylmethylamine (412 μL, 4 mmol) and 4-hydroxy-3-methoxybenzaldehyde (304 mg, 2 mmol) in EtOH (12 mL). Purification by column chromatography (0–6 % MeOH/CH_2_Cl_2_) gave compound **13** as an off-white solid (142 mg, 22 %): ^1^H NMR (500 MHz, [D_6_]DMSO): *δ*=9.21 (br s, 1 H), 8.49 (m, 1 H), 7.73–7.76 (m, 1 H), 7.26–7.29 (m, 1 H), 7.19 (d *J*=8 Hz, 1 H), 6.86 (br s, 1 H), 6.72 (m, 2 H), 5.70 (d, *J*=1.7 Hz, 1 H), 4.79 (d, *J*=16, 1 H), 3.76–3.94 (m, 3 H), 3.72 ppm (s, 3 H); LC–MS: *m*/*z*=317 [*M*+H].

Compounds **7**, **8** and **9** were purchased from ChemDiv.

#### Benzomorpholinone series compounds

**Ethyl 2-(3-oxo-2*H*-benzo[*b*][1,4]oxazin-4(3*H*)-yl)acetate (14)**: 2*H*-benzo[*b*][1,4]oxazin-3(4*H*)-one (100 mg, 0.67 mmol), ethyl bromoacetate (124 mg, 0.74 mmol) and anhydrous K_2_CO_3_ (139 mg, 1.01 mmol) were taken up in DMF (5 mL) and stirred at 60 °C overnight. The reaction mixture was concentrated in vacuo, the residue partitioned with CH_2_Cl_2_ and saturated aqueous NaHCO_3_. The organic layer was dried (MgSO_4_) and concentrated to dryness. Purification by column chromatography (0–5 % MeOH/CH_2_Cl_2_) gave compound **14** as a white solid (132 mg, 84 %): ^1^H NMR (500 MHz, [D_6_]DMSO): *δ*=7.03–7.09 (m, 4 H), 4.73 (s, 2 H), 4.71 (s, 2 H), 4.16 (q, *J*=7.2 Hz, 2 H), 1.21 ppm (t, *J*=7.2 Hz, 3 H); LC–MS: *m*/*z*=236 [*M*+H].

***N***,***N*****-Dimethyl-2-(3-oxo-2*H*-benzo[*b*][1,4]oxazin-4(3*H*)-yl)acetamide (19)**: 2*H*-benzo[*b*][1,4]oxazin-3(4*H*)-one (100 mg, 0.67 mmol), dimethylcarbamic chloride (76 μL, 0.74 mmol) and anhydrous K_2_CO_3_ (139 mg, 1.01 mmol) were treated as described for the synthesis of **14**. After workup a solid was obtained, trituration with Et_2_O gave compound **19** as a white solid (122 mg, 78 %): ^1^H NMR (500 MHz, CDCl_3_): *δ*=6.99–7.02 (m, 3 H), 6.76–7.79 (m, 1 H), 4.70 (s, 2 H), 4.69 (s, 2 H), 3.15 (s, 3 H), 3.02 (s, 3 H); LC–MS: *m*/*z*=235 [*M*+H].

**4-(Benzo[*d*]oxazol-2-ylmethyl)-2*H*-benzo[*b*][1,4]oxazin-3(4*H*)-one (22)**: 2*H*-benzo[*b*][1,4]oxazin-3(4*H*)-one (100 mg, 0.67 mmol), 2-(chloromethyl)benzo[d]oxazole (124 mg, 0.74 mmol) and anhydrous K_2_CO_3_ (139 mg, 1.01 mmol) were treated as described for the synthesis of **14** to give compound **22** as a white solid (148 mg, 79 %): ^1^H NMR (500 MHz, CDCl_3_): *δ*=7.70–7.74 (m, 1 H), 7.50–7.54 (m, 1 H), 7.33–7.37 (m, 2 H), 7.10–7.13 (m, 1 H), 7.02–7.04 (m, 2 H), 6.98–7.02 (m, 1 H), 5.42 (s, 2 H), 4.78 ppm (s, 2 H); LC–MS: *m*/*z*=281 [*M*+H].

**Ethyl 3-(3-oxo-2*H*-benzo[*b*][1,4]oxazin-4(3*H*)-yl)propanoate (16)**: 2*H*-benzo[*b*][1,4]oxazin-3(4*H*)-one (100 mg, 0.67 mmol), ethyl 3-bromopropanoate (134 mg, 0.74 mmol) and anhydrous K_2_CO_3_ (139 mg, 1.01 mmol) were treated as described for the synthesis of **14** to give compound **16** as a white solid (152 mg, 91 %): ^1^H NMR (500 MHz, CDCl_3_): *δ*=7.04–7.06 (m, 2 H), 7.01–7.03 (m, 2 H), 4.61 (s, 2 H), 4.23–4.27 (m, 2 H), 4.15 (q, *J*=7.2 Hz, 2 H), 2.68–2.72 (m, 2 H), 1.26 ppm (t, *J*=7.2 Hz, 3 H); LC–MS: *m*/*z*=250 [*M*+H].

**4-(3-Oxo-3-(pyrrolidin-1-yl)propyl)-2*H*-benzo[*b*][1,4]oxazin-3(4*H*)-one (17)**: Step 1: Ethyl 3-(3-oxo-2*H*-benzo[*b*][1,4]oxazin-4(3*H*)-yl)propanoate (**16**) (2 g, 8.03 mmol) was taken up in THF (100 mL), treated with 2 n aqueous KOH (100 mL), and the reaction was stirred at RT for 1 h. The reaction was then heated at 60 °C, and stirring continued for 2 h. The solvent was removed in vacuo and the aqueous phase was acidified to pH 2 using concentrated HCl. The aqueous phase was then extracted with EtOAc (2×50 mL), and the combined extracts were dried in vacuo to give key intermediate 3-(3-oxo-2H-benzo[*b*][1,4]oxazin-4(3*H*)-yl)propanoic acid (1.7 g, 96 %): ^1^H NMR (500 MHz, [D_6_]DMSO): *δ*=12.4 (br s, 1 H), 7.25 (m, 1 H), 7.07 (m, 1 H), 7.02 (m, 2 H), 4.62 (s, 2 H), 4.14 (q, *J*=7.2 Hz, 2 H), 2.54 ppm (m, 2 H); LC–MS: *m*/*z*=221 [*M*+H].

Step 2: 3-(3-oxo-2*H*-benzo[*b*][1,4]oxazin-4(3*H*)-yl)propanoic acid (200 mg, 0.91 mmol) was taken into CH_2_Cl_2_ (5 mL), added carbonyldiimidazole (162 mg, 1 mmol). Stirred for 15 min then added 1.2 equiv amine (pyrrolidine, 77 mg, 1.09 mmol). Stirred for 18 h at RT. Transferred reaction mixture to a separating funnel, added CH_2_Cl_2_ (30 mL) and washed the organic layer with 1 n HCl (20 mL), 2N NaHCO_3_ (20 mL) and then brine (30 mL). Dried organic layer with MgSO_4_, and removed the solvent in vacuo. Purification by column chromatography (0–6 % MeOH/CH_2_Cl_2_) yielded compound **17** as a white solid (80 mg, 32 %): ^1^H NMR (500 MHz, [D_6_]DMSO): *δ*=7.24 (m, 1 H), 7.06 (m, 1 H), 7.01 (m, 2 H), 4.63 (m, 2 H), 4.11 (m, 2 H), 3.34 (m, 2 H), 3.28 (m, 2 H), 2.57 (m, 2 H), 1.84 (m, 2 H), 1.75 ppm (m, 2 H); LC–MS: *m*/*z*=275 [*M*+H].

**4-(2-Oxo-2-(pyrrolidin-1-yl)ethyl)-2*H*-benzo[*b*][1,4]oxazin-3(4*H*)-one (21)**: The carboxylic acid intermediate was prepared from **14**, as in step 1 for the synthesis of **17**. The free acid was then taken into CH_2_Cl_2_ and reacted with carbonyldiimidazole and pyrrolidine as in step 2 for the synthesis of **17**. Workup and purification as described for the preparation of **17** afforded compound **21** as a white solid (51 %): ^1^H NMR (500 MHz, [D_6_]DMSO): *δ*=6.95–7.03 (m, 4 H), 4.68 (m, 4 H), 3.58 (m, 2 H), 3.32 (m, 2 H), 1.95 (m, 2 H), 1.81 ppm (m, 2 H); LC–MS: *m*/*z*=261 [*M*+H].

***N***,***N*****-Dimethyl-3-(3-oxo-2*H*-benzo[*b*][1,4]oxazin-4(3*H*)-yl)propanamide (18)**: Prepared as described for the synthesis of **17** using 3-(3-oxo-2*H*-benzo[*b*][1,4]oxazin-4(3*H*)-yl)propanoic acid (200 mg, 0.91 mmol) and dimethylamine (1.64 mmol), to give compound **18** as an off-white solid (110 mg, 49 %): ^1^H NMR (500 MHz, [D_6_]DMSO): *δ*=7.22 (m, 1 H), 7.06 (br s, 1 H), 7.02 (br s, 2 H), 4.63 (br s, 2 H), 4.09 (m, 2 H), 2.92 (br s, 3 H), 2.82 (br s, 3 H), 2.63 ppm (br s, 2 H); LC–MS: *m*/*z*=249 [*M*+H].

**4-((5-Methylisoxazol-3-yl)methyl)-2H-benzo[*b*][1,4]oxazin-3(4*H*)-one (23)**: 2*H*-benzo[*b*][1,4]oxazin-3(4*H*)-one (100 mg, 0.67 mmol), 3-(chloromethyl)-5-methylisoxazole (97 mg, 0.74 mmol) and anhydrous K_2_CO_3_ (139 mg, 1.01 mmol) were treated as described for the synthesis of **14**. A solid was obtained by trituration with Et_2_O to give compound **23** as a white solid (162 mg, 99 %): ^1^H NMR (500 MHz, CDCl_3_): *δ*=7.21–7.26 (m, 1 H), 6.98–7.03 (m, 3 H), 5.98–5.99 (m, 1 H), 5.14 (s, 2 H), 4.68 (s, 2 H), 2.38–2.39 ppm (m, 3 H); LC–MS: *m*/*z*=245 [*M*+H].

**4-((2-Methylthiazol-4-yl)methyl)-2*H*-benzo[*b*][1,4]oxazin-3(4*H*)-one (24)**: 2*H*-benzo[*b*][1,4]oxazin-3(4*H*)-one (100 mg, 0.67 mmol), 4-(chloromethyl)-2-methylthiazole hydrochloride (136 mg, 0.74 mmol) and anhydrous K_2_CO_3_ (200 mg, 1.45 mmol) were treated as described for the synthesis of **14** to give compound **24** as an off-white solid (136 mg, 78 %): ^1^H NMR (300 MHz, CDCl_3_): *δ*=7.18–7.24 (m, 1 H), 6.99–7.03 (m, 3 H), 6.95–6.97 (m, 1 H), 5.23 (s, 2 H), 4.69 (s, 2 H), 2.74 ppm (s, 3 H); LC–MS: *m*/*z*=261 [*M*+H].

***N*****-Methyl-2-(3-oxo-2*H*-benzo[*b*][1,4]oxazin-4(3*H*)-yl)acetamide (20)**: Step 1: 2*H*-benzo[*b*][1,4]oxazin-3(4*H*)-one (1.0 g, 6.7 mmol), ethyl bromoacetate (818 mL, 7.4 mmol) and anhydrous K_2_CO_3_ (1.39 g, 10.1 mmol) were taken up in DMF (30 mL) and stirred at 50 °C for 3 h. The reaction mixture was concentrated in vacuo, the residue partitioned with CH_2_Cl_2_ and saturated aqueous NaHCO_3_. The organic layer was collected, dried (MgSO_4_) and concentrated to dryness to give intermediate ethyl 2-(3-oxo-2*H*-benzo[*b*][1,4]oxazin-4(3*H*)-yl)acetate as a clear gum (1.34 g, 5.7 mmol, 85 %): ^1^H NMR (500 MHz, CDCl_3_): *δ*=7.00–7.04 (m, 3 H), 6.75–6.79 (m, 1 H), 4.69 (s, 2 H), 4.66 (s, 2 H), 4.26 (q, *J*=7.2 Hz, 2 H), 1.29 ppm (t, *J*=7.2 Hz, 3 H).

Step 2: Ethyl 2-(3-oxo-2*H*-benzo[*b*][1,4]oxazin-4(3*H*)-yl)acetate (1.34 g, 5.7 mmol) was taken up in THF (20 mL), KOH (640 mg, 11.4 mmol) in water (5 mL) was added and the reaction mixture stirred at 60 °C for 3 h. Solvent was removed in vacuo, the residue acidified to pH 3–4 by addition of 1 m HCl, then extracted into EtOAc. The organic layer was collected, then dried (MgSO_4_) and concentrated to dryness to give intermediate 2-(3-oxo-2*H*-benzo[*b*][1,4]oxazin-4(3*H*)-yl)acetic acid as a white solid (1.1 g, 5.3 mmol, 93 %): ^1^H NMR (300 MHz, [D_6_]DMSO): *δ*=13.11 (s, 1 H), 7.02–7.08 (m, 4 H), 4.71 (s, 2 H), 4.65 ppm (s, 2 H).

Step 3: 2-(3-oxo-2*H*-benzo[*b*][1,4]oxazin-4(3*H*)-yl)acetic acid (200 mg, 0.96 mmol) was taken up in CH_2_Cl_2_ (10 mL), 2 m methylamine in THF (966 μL, 1.92 mmol) and carbonyldiimidazole (172 mg, 1.06 mmol) was added and the reaction mixture was stirred at RT for 18 h. The reaction mixture was then diluted with further CH_2_Cl_2_ (50 mL) and partitioned with saturated aqueous NaHCO_3_. The organic layer was collected, dried (MgSO_4_) and concentrated to dryness. The resulting solid was filtered off and triturated with Et_2_O to give compound **20** as an off-white solid (97 mg, 46 %): ^1^H NMR (300 MHz, [D_6_]DMSO): *δ*=7.16–7.19 (m, 1 H), 7.05–7.08 (m, 2 H), 7.00–7.04 (m, 1 H) 6.18 (br s, 1 H), 4.70 (s, 2 H) 4.54 (s, 2 H), 2.82 ppm (d, *J*=4.9 Hz, 3 H); LC–MS: *m*/*z*=221 [*M*+H].

**4-(Pyridin-2-ylmethyl)-2*H*-benzo[*b*][1,4]oxazin-3(4*H*)-one (25)**: 2*H*-benzo[*b*][1,4]oxazin-3(4*H*)-one (50 mg, 0.34 mmol), 2-(chloromethyl)pyridine hydrochloride (60 mg, 0.37 mmol) and anhydrous K_2_CO_3_ (69 mg, 0.50 mmol) were treated as described for the synthesis of **14** and purified by column chromatography (EtOAc/Hexane) to give compound **25** as a white solid (36 mg, 44 %): ^1^H NMR (500 MHz, CDCl_3_): *δ*=8.58–8.60 (m, 1 H), 7.64 (td, *J*=7.7, 1.8 Hz, 1 H), 7.23–7.26 (m, 1 H), 7.19–7.22 (m, 1 H), 7.03–7.06 (m, 1 H), 6.97–7.02 (m, 2 H), 6.91–6.95 (m, 1 H), 5.28 (s, 2 H), 4.75 ppm (s, 2 H); LC–MS: *m*/*z*=241 [*M*+H].

**4-(Pyridin-4-ylmethyl)-2*H*-benzo[*b*][1,4]oxazin-3(4*H*)-one (26)**: 2*H*-benzo[*b*][1,4]oxazin-3(4*H*)-one (50 mg, 0.34 mmol), 4-(chloromethyl)pyridine hydrobromide (93 mg, 0.37 mmol) and anhydrous K_2_CO_3_ (69 mg, 0.50 mmol) were treated as described for the synthesis of **14** and purified by column chromatography (0–20 % EtOAc/Hexane) to give compound **26** as a white solid (45 mg, 55 %): ^1^H NMR (500 MHz, CDCl_3_): *δ*=8.56–8.59 (m, 2 H), 7.16–7.19 (m, 2 H), 7.05 (dd, *J*=8.0, 1.8 Hz, 1 H), 7.01 (td, *J*=7.6, 1.4 Hz, 1 H), 6.91–6.95 (m, 1 H), 6.75 (dd, *J*=8.1, 1.3 Hz, 1 H), 5.17 (s, 2 H), 4.76 ppm (s, 2 H); LC–MS: *m*/*z*=241 [*M*+H].

**4-((3-Methyl-1,2,4-oxadiazol-5-yl)methyl)-2*H*-benzo[*b*][1,4]oxazin-3(4*H*)-one (27)**: 2*H*-benzo[*b*][1,4]oxazin-3(4*H*)-one (100 mg, 0.67 mmol), 5-(chloromethyl)-3-methyl-1,2,4-oxadiazole (98 mg, 0.74 mmol) and anhydrous K_2_CO_3_ (139 mg, 1.01 mmol) were treated as described for the synthesis of **14** to give compound **27** as an off-white solid (54 mg 33 %): ^1^H NMR (500 MHz, CDCl_3_): *δ*=6.99–7.08 (m, 3 H), 6.86–6.89 (m, 1 H, 5.33 (s, 2 H), 4.74 (s, 2 H), 2.40 ppm (s, 3 H); LC–MS: *m*/*z*=246 [*M*+H].

**4-((5-Methyl-1,2,4-oxadiazol-3-yl)methyl)-2*H*-benzo[*b*][1,4]oxazin-3(4*H*)-one (28)**: 2*H*-benzo[*b*][1,4]oxazin-3(4*H*)-one (100 mg, 0.67 mmol), 3-(chloromethyl)-5-methyl-1,2,4-oxadiazole (98 mg, 0.74 mmol) and anhydrous K_2_CO_3_ (139 mg, 1.01 mmol) were treated as described for the synthesis of **14** to give compound **28** as a white solid (135 mg, 82 %): ^1^H NMR (300 MHz, CDCl_3_): *δ*=6.99–7.04 (m, 4 H), 5.24 (s, 2 H), 4.72 (s, 2 H), 2.58 ppm (s, 3 H); LC–MS: *m*/*z*=246.0 [*M*+H].

**4-((5-Isobutyl-1,2,4-oxadiazol-3-yl)methyl)-2*H*-benzo[*b*][1,4]oxazin-3(4*H*)-one (29)**: 2*H*-benzo[*b*][1,4]oxazin-3(4*H*)-one (100 mg, 0.67 mmol), 3-(chloromethyl)-5-isobutyl-1,2,4-oxadiazole (129 mg, 0.74 mmol) and anhydrous K_2_CO_3_ (139 mg, 1.01 mmol) were treated as described for the synthesis of **14** to give compound **29** as a white solid (162 mg, 84 %): ^1^H NMR (500 MHz, CDCl_3_): *δ*=6.98–7.03 (m, 4 H), 5.25 (s, 2 H), 4.72 (s, 2 H), 2.75 (d, *J*=7.2 Hz, 2 H), 2.19 (hept, *J*=6.8 Hz, 1 H), 0.98 ppm (d, *J*=6.7 Hz, 6 H); LC–MS: *m*/*z*=288 [*M*+H].

**4-((5-Isobutylisoxazol-3-yl)methyl)-2*H*-benzo[*b*][1,4]oxazin-3(4*H*)-one (30)**: 2*H*-benzo[*b*][1,4]oxazin-3(4*H*)-one (100 mg, 0.67 mmol), 3-(chloromethyl)-5-isobutylisoxazole (128 mg, 0.74 mmol) and anhydrous K_2_CO_3_ (139 mg, 1.01 mmol) were treated as described for the synthesis of **14** to give compound **30** as an off-white solid (165 mg, 86 %): ^1^H NMR (500 MHz, CDCl_3_): *δ*=7.21–7.25 (m, 1 H), 6.98–7.03 (m, 3 H), 5.97 (s, 1 H), 5.15 (s, 2 H), 4.68 (s, 2 H), 2.58 (d, *J*=7.1 Hz, 2 H), 2.01 (hept, *J*=6.8 Hz, 1 H), 0.93 ppm (d, *J*=6.6 Hz, 6 H); LC–MS: *m*/*z*=287 [*M*+H].

**4-((6-Methylpyridin-2-yl)methyl)-2H-benzo[*b*][1,4]oxazin-3(4*H*)-one (31)**: 2*H*-benzo[*b*][1,4]oxazin-3(4*H*)-one (50 mg, 0.34 mmol), 2-(bromomethyl)-6-methylpyridine (69 mg, 0.37 mmol) and anhydrous K_2_CO_3_ (69 mg, 0.50 mmol) were treated as described for the synthesis of **14** to give compound **31** as a white solid (71 mg, 82 %): ^1^H NMR (500 MHz, CDCl_3_): *δ*=7.50 (t, *J*=7.7 Hz, 1 H), 7.04–7.06 (m, 1 H), 6.95–7.02 (m, 4 H), 6.91–6.95 (m, 1 H), 5.24 (s, 2 H), 4.74 (s, 2 H), 2.58 ppm (s, 3 H); LC–MS: *m*/*z*=255 [*M*+H].

**4-((1-Methyl-1*H*-pyrazol-3-yl)methyl)-2*H*-benzo[*b*][1,4]oxazin-3(4*H*)-one (32)**: Step 1: Thionyl chloride (0.5 mL, excess) was added to a solution of (1-methyl-1*H*-pyrazol-3-yl)methanol (100 mg, 0.89 mmol) in CHCl_3_ and heated at 60 °C for 2 h. The solvent was removed in vacuo and the resulting crude 3-(chloromethyl)-1-methyl-1*H*-pyrazole hydrochloride material used without purification in the next step: LC–MS: *m*/*z*=131.1 [*M*+H].

Step 2: 2*H*-benzo[*b*][1,4]oxazin-3(4*H*)-one (111 mg, 0.74 mmol), crude 3-(chloromethyl)-1-methyl-1*H*-pyrazole hydrochloride (assumed 0.89 mmol) and anhydrous K_2_CO_3_ (300 mg, 2.17 mmol) were treated as described for the synthesis of **14** to give compound **32** as a gum (170 mg, 94 % over two steps): ^1^H NMR (500 MHz, CDCl_3_): *δ*=7.30–7.33 (m, 1 H), 7.26–7.27 (m, 1 H), 6.96–7.01 (m, 3 H), 6.17 (d, *J*=2.2 Hz, 1 H), 5.12 (s, 2 H), 4.67 (s, 2 H), 3.88 ppm (s, 3 H); LC–MS: *m*/*z*=244 [*M*+H].

**Ethyl 2-(3-oxo-2*H*-benzo[*b*][1,4]oxazin-4(3*H*)-yl)propanoate (15)**: 2*H*-benzo[*b*][1,4]oxazin-3(4*H*)-one (100 mg, 0.67 mmol), ethyl 2-bromopropanoate (96 μL, 0.74 mmol) and anhydrous K_2_CO_3_ (139 mg, 1.01 mmol) were treated as described for the synthesis of **14** then purified by column chromatography (0–20 % EtOAc/hexane) to give compound **15** as an off-white solid (100 mg, 60 %): ^1^H NMR (300 MHz, CDCl_3_): *δ*=6.99–7.05 (m, 3 H), 7.83–7.86 (m, 1 H), 5.36 (q, *J*=7.1 Hz, 1 H), 4.62 (q, *J*=16.5 Hz, 2 H), 4.18–4.25 (m, 2 H), 1.64 (d, *J*=7.1 Hz, 3 H), 1.20 ppm (t, *J*=7.1 Hz, 3 H); LC–MS: *m*/*z*=250 [*M*+H].

**8-Bromo-4-((5-methylisoxazol-3-yl)methyl)-2*H*-benzo[*b*][1,4]oxazin-3(4*H*)-one (38)**: Step 1: 2-chloroacetyl chloride (1.7 mL, 21.3 mmol) was added dropwise to a mixture of 2-amino-6-bromophenol (4 g, 21.3 mmol) and K_2_CO_3_ (2.94 g, 21.3 mmol) in DMF (40 mL). The reaction mixture was heated at 80 °C for 3 h then the solvent removed in vacuo. The residue was partitioned between CH_2_Cl_2_ and water, the organic layer was collected, dried with MgSO_4_ and the solvent was then removed in vacuo. The resulting solid was filtered off and triturated with Et_2_O to give intermediate 8-bromo-2*H*-benzo[*b*][1,4]oxazin-3(4*H*)-one as an off-white solid (1.87 g, 39 %): ^1^H NMR (500 MHz, CDCl_3_): *δ*=8.67 (br s, 1 H), 7.23 (dd, *J*=8.1, 1.5 Hz, 1 H) 6.86 (t, *J*=8.0 Hz, 1 H), 6.79 (dd, *J*=7.9, 1.5 Hz, 1 H), 4.74 ppm (s, 2 H); LC–MS: *m*/*z*=226/228 [*M*+H].

Step 2: 8-bromo-2*H*-benzo[*b*][1,4]oxazin-3(4*H*)-one (200 mg, 0.88 mmol), 3-(chloromethyl)-5-methylisoxazole (128 mg, 0.97 mmol) and anhydrous K_2_CO_3_ (183 mg, 1.33 mmol) were treated as described for the synthesis of **14** to give compound **38** as a pale-yellow solid (252 mg, 89 %): ^1^H NMR (500 MHz, CDCl_3_): *δ*=7.24 (dd, *J*=8.2, 1.4 Hz, 1 H), 7.20 (dd, *J*=8.2, 1.3 Hz, 1 H), 6.89 (t, *J*=8.2 Hz, 1 H), 5.98 (d, *J*=0.8 Hz, 1 H), 5.13 (s, 2 H), 4.77 (s, 2 H), 2.39 ppm (d, *J*=0.8 Hz, 3 H); LC–MS: *m*/*z*=322/324 [*M*+H].

**2-Methyl-4-((5-methylisoxazol-3-yl)methyl)-2*H*-benzo[*b*][1,4]oxazin-3(4*H*)-one (34)**: Step 1: 2-chloropropanoyl chloride (4.45 mL, 46 mmol) was added dropwise to a mixture of 2-aminophenol (5 g, 46 mmol) and K_2_CO_3_ (6.33 g, 46 mmol) in DMF (50 mL) at 0 °C. The reaction mixture was heated at 60 °C for 2 h then the solvent removed in vacuo. The residue was partitioned between CH_2_Cl_2_ and water, the organic layer was collected, dried with MgSO_4_ and the solvent was then removed in vacuo. The resulting solid was filtered and triturated with Et_2_O to give intermediate 2-methyl-2*H*-benzo[*b*][1,4]oxazin-3(4*H*)-one as a colourless gum (4.25 g, 57 %): ^1^H NMR (500 MHz, CDCl_3_): *δ*=8.69 (br s, 1 H), 6.95–7.02 (m, 3 H) 6.82–6.86 (m, 1 H), 4.68 (q, *J*=6.9 Hz, 1 H), 1.60 ppm (d, *J*=6.8 Hz, 3 H); LC–MS: *m*/*z*=164 [*M*+H].

Step 2: 2-methyl-2*H*-benzo[*b*][1,4]oxazin-3(4*H*)-one (100 mg, 0.61 mmol), 3-(chloromethyl)-5-methylisoxazole (89 mg, 0.68 mmol) and anhydrous K_2_CO_3_ (127 mg, 0.92 mmol) were treated as described for the synthesis of **14**, then purified by column chromatography (0–20 % EtOAc/hexane) to give compound **34** as an off-white solid (140 mg, 89 %): ^1^H NMR (500 MHz, CDCl_3_): *δ*=7.18–7.21 (m, 1 H), 6.98–7.02 (m, 3 H), 5.95–5.96 (m, 1 H), 5.18 (d, *J*=15.7 Hz, 1 H), 5.08 (d, *J*=15.7 Hz, 1 H), 4.69 (q, *J*=6.8 Hz, 1 H), 2.38 (d, *J*=0.9 Hz, 3 H), 1.60 ppm (d, *J*=6.8 Hz, 3 H); LC–MS: *m*/*z*=259 [*M*+H].

**7-Bromo-4-((5-methylisoxazol-3-yl)methyl)-2*H*-benzo[*b*][1,4]oxazin-3(4*H*)-one (37)**: Step 1: 2-chloroacetyl chloride (2.33 mL, 29.3 mmol), 2-amino-5-bromophenol (5 g, 26.6 mmol) and K_2_CO_3_ (4.41 g, 31.9 mmol) were treated as described in step 1 for the synthesis of **38** to give intermediate 7-bromo-2*H*-benzo[*b*][1,4]oxazin-3(4*H*)-one as an off-white solid. The product was purified as described in step 1 of the synthesis of compound **38** (4.90 g, 81 %): ^1^H NMR (500 MHz, CDCl_3_): *δ*=7.91 (br s, 1 H), 7.15–7.16 (m, 1 H) 7.10 (dd, *J*=8.4, 2.1 Hz, 1 H), 6.68 (d, *J*=8.4 Hz, 1 H), 4.63 ppm (s, 2 H); LC–MS: *m*/*z*=226/228 [*M*+H].

Step 2: 7-bromo-2*H*-benzo[*b*][1,4]oxazin-3(4*H*)-one (4.19 g, 19 mmol), 3-(chloromethyl)-5-methylisoxazole (2.68 g, 20 mmol) and anhydrous K_2_CO_3_ (3.84 g, 28 mmol) were treated as described for the synthesis of **14** to give compound **37** as a white solid (4.60 g, 77 %): ^1^H NMR (500 MHz, CDCl_3_): *δ*=7.14–7.15 (m, 1 H), 7.11–7.14 (m, 1 H), 7.08–7.11 (m, 1 H), 5.97–5.98 (m, 1 H), 5.11 (s, 2 H), 4.67 (s, 2 H), 2.39 ppm (d, *J*=0.8 Hz, 3 H); LC–MS: *m*/*z*=322/324 [*M*+H].

**6-Chloro-4-((5-methylisoxazol-3-yl)methyl)-2*H*-benzo[*b*][1,4]oxazin-3(4*H*)-one (35)**: Step 1: 2-amino-4-chlorophenol (1.44 g, 10 mmol), 2-bromoacetylbromide (3.03 g, 15 mmol) and K_2_CO_3_ (1.66 g, 12 mmol) were treated as described in step 1 for the synthesis of **38** to give intermediate 6-chloro-2*H*-benzo[*b*][1,4]oxazin-3(4*H*)-one (1.36 g, 74 %): LC–MS: *m*/*z*=185 [*M*+H].

Step 2: 6-chloro-2*H*-benzo[*b*][1,4]oxazin-3(4*H*)-one (53 mg, 0.29 mmol), 3-(chloromethyl)-5-methylisoxazole (42 mg, 0.32 mmol) and anhydrous K_2_CO_3_ (60 mg, 0.44 mmol) were treated as described for the synthesis of **14** to give compound **35** as an off-white solid (57 mg, 71 %): ^1^H NMR (500 MHz, [D_6_]DMSO): *δ*=7.23 (m, 1 H), 7.07 (m, 2 H), 6.16 (br s, 1 H), 5.17 (s, 2 H), 4.77 (s, 2 H), 2.37 ppm (s, 3 H); LC–MS: *m*/*z*=280/281 [*M*+H].

**7-Chloro-4-((5-methylisoxazol-3-yl)methyl)-2*H*-benzo[*b*][1,4]oxazin-3(4*H*)-one (36)**: Step 1: 2-amino-5-chlorophenol (1.44 g, 10 mmol), 2-bromoacetylbromide (3.03 g, 15 mmol) and K_2_CO_3_ (1.66 g, 12 mmol) were treated as described in step 1 for the synthesis of **38** to give intermediate 7-chloro-2*H*-benzo[*b*][1,4]oxazin-3(4*H*)-one (1.29 g, 70 %): LC–MS: *m*/*z*=185 [*M*+H].

Step 2: 7-chloro-2*H*-benzo[*b*][1,4]oxazin-3(4*H*)-one (35 mg, 0.24 mmol), 3-(chloromethyl)-5-methylisoxazole (32 mg, 0.24 mmol) and anhydrous K_2_CO_3_ (40 mg, 0.37 mmol) were treated as described for the synthesis of **14** to give compound **36** as an off-white solid (38 mg, 57 %): ^1^H NMR (500 MHz, [D_6_]DMSO): *δ*=7.16 (m, 1 H), 7.12 (m, 2 H), 6.16 (br s, 1 H), 5.15 (s, 2 H), 4.80 (s, 2 H), 2.36 ppm (s, 3 H); LC–MS: *m*/*z*=280/281 [*M*+H].

**4-((5-Methylisoxazol-3-yl)methyl)-8-(4-(piperidin-4-yl)butyl)-2*H*-benzo[*b*][1,4]oxazin-3(4*H*)-one (39)**: Step 1: *tert*-butyl 4-(but-3-en-1-yl)piperidine-1-carboxylate (334 mg, 1.40 mmol), 9-BBN (0.5 m in THF, 3.35 mL, 1.68 mmol) and **38** (300 mg, 0.93 mmol) were treated as described in step 1 for the synthesis of **40**. The Suzuki reaction was carried out at 90 °C for 1 h, then at 110 °C for 30 min, to give intermediate *tert*-butyl 4-(4-(4-((5-methylisoxazol-3-yl)methyl)-3-oxo-3,4-dihydro-2*H*-benzo[*b*][1,4]oxazin-8-yl)butyl)piperidine-1-carboxylate: LC–MS: *m*/*z*=484 [*M*+H].

Step 2: *tert*-butyl 4-(4-(4-((5-methylisoxazol-3-yl)methyl)-3-oxo-3,4-dihydro-2*H*-benzo[*b*][1,4]oxazin-8-yl)butyl)piperidine-1-carboxylate was taken up in CH_2_Cl_2_ (10 mL), trifluoroacetic acid (1 mL, excess) was added dropwise and the reaction mixture was stirred at RT for 3 h. The solvent was removed in vacuo, the residue was taken into CH_2_Cl_2_ (30 mL) then partitioned between CH_2_Cl_2_ and saturated aqueous NaHCO_3_. The organic layer was collected, dried (MgSO_4_) and concentrated in vacuo. The solid obtained was filtered off and triturated with Et_2_O to give compound **39** as a white solid (61 mg, 17 % over two steps): ^1^H NMR (500 MHz, [D_6_]DMSO): *δ*=6.98 (dd, *J*=7.8, 1.9 Hz, 1 H), 6.94 (t, *J*=7.6 Hz, 1 H), 6.91 (dd, *J*=7.2, 1.9 Hz, 1 H), 6.14 (s, 1 H), 5.13 (s, 2 H), 4.74 (s, 2 H), 2.87–2.94 (m, 2 H), 2.55–2.60 (m, 2 H), 2.37 (s, 3 H), 1.75–1.83 (m, 2 H), 1.54–1.62 (m, 2 H), 1.47–1.54 (m, 2 H), 1.27–1.34 (m, 2 H), 1.13–1.25 (m, 3 H), 0.91–1.10 ppm (m, 2 H); LC–MS: *m*/*z*=384 [*M*+H].

**4-(4-(4-((5-Methylisoxazol-3-yl)methyl)-3-oxo-3,4-dihydro-2*H*-benzo[*b*][1,4]oxazin-8-yl)butyl)homopiperazine-1-methyl (40)**: Step 1: Following the methodology reported by Billotte,[17] a solution of *tert*-butyl 4-(but-3-en-1-yl)homopiperazine-1-carboxylate (775 mg, 3.1 mmol) in THF (2 mL), under argon at RT, was treated dropwise with 9-BBN (0.5 m in THF, 7.7 mL, 3.9 mmol). The reaction mixture was heated at 90 °C in the microwave for 30 min. The resulting solution was then transferred into a stirred mixture of **38** (500 mg, 1.55 mmol) and 2 m potassium phosphate solution (1.5 mL, 3.1 mmol) in DMF (2 mL) and water (1 mL) under argon. After bubbling argon through the reaction for 5 min at RT, Pd(PPh_3_)_4_ (90 mg, 5 mol %) was added, the reaction vessel sealed and heated at 90 °C in the microwave for 120 min. The reaction mixture was then concentrated in vacuo, diluted with CH_2_Cl_2_ and aqueous ammonia solution, the organic layer was washed with brine, then dried (MgSO_4_) and concentrated in vacuo. Column chromatography with 0–10 % MeOH/(0.1 %) NH_3_/CH_2_Cl_2_ eluent, followed by freeze drying, gave intermediate *tert*-butyl 4-(4-(4-((5-methylisoxazol-3-yl)methyl)-3-oxo-3,4-dihydro-2*H*-benzo[*b*][1,4]oxazin-8-yl)butyl)homopiperazine-1-carboxylate (200 mg, 0.4 mmol, 26 %): LC–MS: *m*/*z*=499 [*M*+H].

Step 2: *tert*-butyl 4-(4-(4-((5-methylisoxazol-3-yl)methyl)-3-oxo-3,4-dihydro-2*H*-benzo[*b*][1,4]oxazin-8-yl)butyl)homopiperazine-1-carboxylate (200 mg, 0.4 mmol) was stirred overnight at RT with trifluoroacetic acid (2 mL) in CH_2_Cl_2_ (10 mL). The reaction mixture was then concentrated in vacuo, and taken up in MeOH (10 mL). The product was purified using an SCX column, eluting with 2 n NH_3_/MeOH, followed by silica chromatography with 0–10 % MeOH/(0.1 %) NH_3_/CH_2_Cl_2_ eluent to give intermediate 4-(4-(4-((5-methylisoxazol-3-yl)methyl)-3-oxo-3,4-dihydro-2*H*-benzo[*b*][1,4]oxazin-8-yl)butyl)homopiperazine (95 mg, 0.24 mmol, 60 %): LC–MS: *m*/*z*=399 [*M*+H].

Step 3: 4-(4-(4-((5-methylisoxazol-3-yl)methyl)-3-oxo-3,4-dihydro-2*H*-benzo[*b*][1,4]oxazin-8-yl)butyl)homopiperazine (54 mg, 0.14 mmol) was stirred in CHCl_3_ with paraformaldehyde (5 equiv) for 1 h at RT. Sodium cyanoborohydride (5 equiv) was then added and the reaction stirred overnight at RT. A further 2 equivalents of paraformaldehyde and sodium cyanoborohydride were added and the reaction stirred for a further 18 h: LC–MS showed product at *m*/*z*=413 [*M*+H]. The product was recovered by SCX purification, eluting with 2 n NH_3_/MeOH. The solvent was removed in vacuo to give the title compound (**40**) as an off-white solid (50 mg, 0.12 mmol, 86 %): ^1^H NMR (500 MHz, CDCl_3_): *δ*=7.06 (dd, *J*=8, 1.5 Hz, 1 H), 6.86–6.93 (m, 2 H), 5.98 (d, *J*=0.8 Hz, 1 H), 5.12 (s, 2 H), 4.74 (s, 2 H), 4.65 (s, 2 H), 2.71 (m, 4 H), 2.63 (m, 5 H), 2.49 (t, 7.5 Hz, 2 H), 2.38 (d, *J*=0.8 Hz, 2 H), 2.37 (s, 3 H), 1.82 (m, 2 H), 1.54 ppm (m, 4 H); LC–MS: *m*/*z*=413 [*M*+H].

**4-((5-Methylisoxazol-3-yl)methyl)-8-(4-(1-methylpiperidin-4-yl)butyl)-2*H*-benzo[*b*][1,4]oxazin-3(4*H*)-one (41)**: 4-(but-3-en-1-yl)-1-methylpiperidine (107 mg, 0.70 mmol), 9-BBN (0.5 m in THF, 0.86 mL, 0.93 mmol) and **38** (150 mg, 0.47 mmol) were treated as described in step 1 for the synthesis of **40** to give compound **41** as an off-white solid (65 mg, 35 %): ^1^H NMR (500 MHz, [D_6_]DMSO): *δ*=7.09 (m, 1 H), 6.94 (m, 1 H), 6.89 (m, 1 H), 6.00 (br s, 1 H), 5.14 (s, 2 H), 4.67 (s, 2 H), 2.84 (m, 2 H), 2.62 (m, 2 H), 2.40 (s, 3 H), 2.27 (s, 3 H), 1.86–1.91 (m, 2 H), 1.53–1.70 (m, 6 H), 1.35 (m, 2 H), 1.21–1.31 ppm (m, 3 H); LC–MS: *m*/*z*=398 [*M*+H].

**(*R*)-4-((5-Methylisoxazol-3-yl)methyl)-7-(3-((1-methylpyrrolidin-3-yl)methoxy)propyl)-2*H*-benzo[*b*][1,4]oxazin-3(4*H*)-one (42)**: Step 1: (*R*)-*tert*-butyl 3-((allyloxy)methyl)pyrrolidine-1-carboxylate (225 mg, 0.93 mmol), 9-BBN (0.5 m in THF, 2.36 mL, 1.18 mmol) and **37** (200 mg, 0.62 mmol) were treated as described in step 1 for the synthesis of **40**. The Suzuki reaction was carried out at 120 °C for 90 min, to give (*R*)-*tert*-butyl 3-((3-(4-((5-methylisoxazol-3-yl)methyl)-3-oxo-3,4-dihydro-2*H*-benzo[*b*][1,4]oxazin-7-yl)propoxy)methyl)pyrrolidine-1-carboxylate, which was used without further purification: LC–MS: *m*/*z*=486 [*M*+H].

Step 2: (*R*)-*tert*-butyl 3-((3-(4-((5-methylisoxazol-3-yl)methyl)-3-oxo-3,4-dihydro-2*H*-benzo[*b*][1,4]oxazin-7-yl)propoxy)methyl)pyrrolidine-1-carboxylate (assumed 0.62 mmol) was deprotected as described for the synthesis of **39** to give (*R*)-4-((5-methylisoxazol-3-yl)methyl)-7-(3-(pyrrolidin-3-ylmethoxy)propyl)-2*H*-benzo[*b*][1,4]oxazin-3(4*H*)-one, which was used without further purification: LC–MS: *m*/*z*=386 [*M*+H].

Step 3: (*R*)-4-((5-methylisoxazol-3-yl)methyl)-7-(3-(pyrrolidin-3-ylmethoxy)propyl)-2*H*-benzo[*b*][1,4]oxazin-3(4*H*)-one (assumed 0.62 mmol) was taken up in dry CHCl_3_ (5 mL), paraformaldehyde (93 mg, 3.10 mmol) and sodium triacetoxyborohydride (657 mg, 3.10 mmol) added and the reaction mixture stirred overnight at RT. Water (5 mL) was added, the layers separated and the organics concentrated to dryness and purified by column chromatography 0–10 % MeOH/(0.1 %) NH_3_/CH_2_Cl_2_ eluent to give compound **42** as a white solid (63 mg, 25 % over three steps): ^1^H NMR (500 MHz, [D_6_]DMSO): *δ*=7.09–7.13 (m, 1 H), 6.79–7–6.84 (m, 2 H), 5.97–5.98 (m, 1 H), 5.12 (s, 2 H), 4.65 (s, 2 H), 3.54–3.59 (m, 1 H), 3.40–3.46 (m, 3 H), 3.20–3.25 (m, 1 H), 2.57–2.67 (m, 3 H), 2.52 (s, 2 H), 2.34–2.41 (m, 1 H), 2.37–2.38 (m, 3 H), 1.95–2.03 (m, 2 H), 1.81–1.90 (m, 3 H), 1.73–1.81 (m, 1 H), 1.63–1.71 ppm (m, 1 H); LC–MS: *m*/*z*=400 [*M*+H].

**4-((5-Methylisoxazol-3-yl)methyl)-7-(3-(piperidin-4-yloxy)propyl)-2*H*-benzo[*b*][1,4]oxazin-3(4*H*)-one (43)**: Step 1: 4-hydroxy-*N*-Boc-piperdine (3.02 g, 15 mmol) was taken into THF (150 mL) and cooled to 0 °C. NaH (60 % suspension on mineral oils) (900 mg, 22.5 mmol) was added slowly, and the reaction was warmed to RT (10 min) then cooled back to 0 °C. Allyl bromide (2 g, 16.5 mmol) was added and the reaction was stirred for 18 h at RT. The reaction was diluted with EtOAc (80 mL) and transferred to separating funnel. The organic layer was washed with water (50 mL), brine (50 mL) then dried (MgSO_4_) and concentrated in vacuo. Purification by column chromatography (5–30 % EtOAc/pet ether) yielded intermediate *tert*-butyl 4-(allyloxy)piperidine-1-carboxylate (1.52 g, 72 %): ^1^H NMR (500 MHz, CDCl_3_): *δ*=5.95 (m, 1 H), 5.31 (m, 1 H), 5.20 (m, 1 H), 4.04 (d, *J*=5.7, 2 H), 3.80 (s, 2 H), 3.52 (m, 1 H), 3.10 (m, 2 H), 1.85 (m, 2 H), 1.54 (m, 2 H), 1.48 ppm (s, 9 H); LC–MS: *m*/*z*=142 [*M*+H].

Step 2: *tert*-butyl 4-(allyloxy)piperidine-1-carboxylate (219 mg, 1.55 mmol), 9-BBN (0.5 m in THF, 3.4 mL, 1.7 mmol) and **37** (150 mg, 0.47 mmol) were treated as described in step 1 for the synthesis of **40**. Purification by column chromatography (5–30 % EtOAc/pet ether) gave intermediate tert-butyl 4-(2-(4-((5-methylisoxazol-3-yl)methyl)-3-oxo-3,4-dihydro-2*H*-benzo[*b*][1,4]oxazin-7-yl)ethoxy)piperidine-1-carboxylate (184 mg, 0.39 mmol): LC–MS: *m*/*z*=472 [*M*+H].

Step 3: *tert*-butyl 4-(2-(4-((5-methylisoxazol-3-yl)methyl)-3-oxo-3,4-dihydro-2*H*-benzo[*b*][1,4]oxazin-7-yl)ethoxy)piperidine-1-carboxylate (40 mg, 0.085 mmol) was taken into CH_2_Cl_2_ (6 mL), trifluoroacetic acid (1 mL) was added and the reaction was stirred at RT for 2 h. Solvent was removed in vacuo, and the residue was dissolved up in MeOH (10 mL) and purified on an SCX column, elution with 2 n NH_3_/MeOH. This yielded compound **43** as an off-white solid (25 mg, 73 %): ^1^H NMR (500 MHz, [D_6_]DMSO): *δ*=6.79 (m, 1 H), 6.63 (m, 2 H), 5.89 (br s, 1 H), 5.20 (br s, 2 H), 4.88 (s, 2 H), 4.48 (s, 2 H), 3.60 (br s, 1 H), 3.04–3.16 (m, 4 H), 2.65 (m, 1 H), 2.30 (m, 2 H), 2.15 (m, 2 H), 2.12 (s, 3 H), 1.50 (m, 2 H), 0.98 ppm (m, 2 H); LC–MS: *m*/*z*=372 [*M*+H].

**4-((5-Methylisoxazol-3-yl)methyl)-7-(4-(1-methylpiperidin-4-yl)butyl)-2*H*-benzo[*b*][1,4]oxazin-3(4*H*)-one (44)**: 4-(but-3-en-1-yl)-1-methylpiperidine (107 mg, 0.70 mmol), 9-BBN (0.5 m in THF, 0.86 mL, 0.93 mmol) and **37** (150 mg, 0.47 mmol) were treated as described in step 1 for the synthesis of compound **40** to give compound **44** as a white solid (65 mg, 35 %): ^1^H NMR (500 MHz, [D_6_]DMSO): *δ*=7.01–7.04 (m, 1 H), 6.86–6.87 (m, 1 H), 6.83–6.86 (m, 1 H), 6.13–6.14 (m, 1 H), 5.12 (s, 2 H), 4.72 (s, 2 H), 2.68–2.73 (m, 2 H), 2.48–2.51 (m, 2 H), 2.37 (d, *J*=0.6 Hz, 3 H), 2.12 (s, 3 H), 1.74–1.80 (m, 2 H), 1.54–1.60 (m, 2 H), 1.24–1.31 (m, 2 H), 1.17–1.23 (m, 2 H), 1.04–1.14 ppm (m, 3 H); LC–MS: *m*/*z*=398 [*M*+H].

### Protein crystallography

*Lm*NMT protein–ligand complexes were determined using methods described previously.[9] Diffraction data were measured either in-house using a rotating anode source (Rigaku Micromax 007) equipped with an image plate detector (R-AXIS IV^++^) or at synchrotron sources (ESRF beamline ID14–1 and Diamond beamline I03). Coordinates for *Lm*NMT–ligand complexes and associated diffraction data have been deposited in the RCSB Protein Data Bank (PDB) with accession codes 5AG5, 5AG4, 5AG6, 5AG7, and 5AGE for compounds **6**, **7**, **13**, **14**, and **44** respectively.

### Virtual screening

**Preparation of small-molecule database**: A subset of the Drug Discovery Unit Dundee in-house database containing 2.2 million commercially available compounds was created using the criteria:[18]

- No unwanted groups;
- No primary and secondary amides, or sulfonamides to avoid high polar surface area;
- Number of heavy atoms between 10 and 2;
- clog*P* in the range of 0–4;
- Fewer than five ring systems;
- Fewer than eight rotatable bonds;
- Fewer than seven HBAs, fewer than four HBDs;
- Sum of HBAs and HBDs >1 but <9;
- No fused ring systems.

In total, 145 127 molecules were contained in this subset. Subsequently, protonation states and tautomers were calculated using in-house scripts based on OpenEye's python toolkit (OEChem, version 1.7.2.4, OpenEye Scientific Software Inc., Santa Fe, NM (USA), 2009 (http://www.eyesopen.com), whereas for acidic groups with estimated p*K*_a_ values between 5 and 9, both the neutral and charged forms were stored. Corina (Molecular Networks, Germany) was used to generate three-dimensional coordinates from smiles.

**Pharmacophore search**: The module UNITY in SYBYL 8.0 was applied to perform a flexible 3D pharmacophore search. The crystal structure of *Lm*NMT in complex with a pyrazole sulfonamide (PDB code: 4A30) was used as receptor. Acceptor atoms, donor sites, aromatic features, and exclusion volumes were selected to create the UNITY query as described in the *Results and Discussion* section (Figure 3). The UNITY database was created by uploading the mol2 file that was created by Corina. The import options “perceive chirality at carbons”, “perceive chirality at nitrogen and phosphorous”, and “perceive chirality at double bonds” were chosen; 8773 compounds passed the pharmacophore filter and were subsequently clustered using the Jarvis–Patrick algorithm of the Daylight clustering package (Daylight, Aliso Viejo, CA, USA). Representative hits per cluster were minimised in the binding site using the Moloc MAB force field.[19] The binding modes obtained were visually inspected, and a list of 200 compounds was selected for purchase.

### Drug metabolism and pharmacokinetics (DMPK)

*In vitro intrinsic clearance assay (CL*_int_ *(m))*: Test compound (0.5 μm) was incubated with female CD1 mouse liver microsomes (Xenotech LLC; 0.5 mg (mL 50 mm potassium phosphate buffer)^−1^, pH 7.4), and the reaction was started by the addition of excess NADPH (8 mg (mL 50 mm potassium phosphate buffer)^−1^, pH 7.4). Immediately, at time zero, then at 3, 6, 9, 15 and 30 min, an aliquot (50 μL) of the incubation mixture was removed and mixed with acetonitrile (100 μL) to stop the reaction. Internal standard was added to all samples, the samples were centrifuged (10 min, 5 °C, 3270 *g*) to sediment precipitated protein and the plates then sealed prior to UPLC–MS/MS analysis using a Quattro Premier XE (Waters Corp., USA).

XLfit (IDBS, UK) was used to calculate the exponential decay and consequently the rate constant (*k*) from the ratio of peak area of test compound to internal standard at each time point. The rate of intrinsic clearance (*CL*_int_: [mL min^−1^ (g liver)^−1^]) of each test compound was then calculated using Equation (1), in which *V* [mL (mg protein)^−1^] is the incubation volume per mg protein added, and microsomal protein yield is taken as 52.5 mg protein per gram of liver. Verapamil (0.5 μm) was used as a positive control to confirm acceptable assay performance.


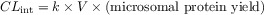
(1)

*In vitro plasma protein binding experiments*: In brief, a 96-well equilibrium dialysis apparatus was used to determine the free fraction in plasma for each compound (HT Dialysis LLC, Gales Ferry, CT, USA). Membranes (12–14 kDa cutoff) were conditioned in deionised water for 60 min, followed by conditioning in 80:20 deionised water/EtOH for 20 min, and then rinsed in isotonic buffer before use. Female CD1 mouse plasma was removed from the freezer and allowed to thaw on the day of experiment. Thawed plasma was then centrifuged (Allegra X12-R, Beckman Coulter, USA, 10 min, 5 °C, 3270 *g*), spiked with test compound (10 μg g^−1^), and 150 μL aliquots (*n*=6 replicate determinations) loaded into the 96-well equilibrium dialysis plate. Dialysis versus isotonic buffer (150 μL) was carried out for 5 h in a temperature-controlled incubator at ∼37 °C (Barworld Scientific Ltd., UK) using an orbital microplate shaker at 125 rpm (Barworld Scientific). At the end of the incubation period, aliquots of plasma or buffer were transferred to micronic tubes (Micronic B.V., the Netherlands), and the composition in each tube was balanced with control fluid such that the volume of buffer to plasma was the same. Sample extraction was performed by the addition of 400 μL acetonitrile containing an appropriate internal standard. Samples were allowed to mix for 1 min and then centrifuged at 3000 rpm in 96-well blocks for 15 min (Allegra X12-R, Beckman Coulter, USA). All samples were analysed by UPLC–MS/MS on a Quattro Premier XE Mass Spectrometer (Waters Corp., USA). The unbound fraction was determined as the ratio of the peak area in buffer to that in plasma.

*Mouse brain penetration studies*: Compounds **41** and **44** were dosed as a bolus solution intravenously at 1 mg free base per kg (dose volume: 5 mL kg^−1^; dose vehicle: 10 % DMSO, 95 % saline or 10 % DMSO, 40 % PEG400, 50 % saline) to female NMRI mice (*n*=6). At 5 and 30 min (compound **41** only) following intravenous bolus injection of test compound, mice (*n*=3 per time point) were placed under terminal anaesthesia with isofluorane. A blood sample was taken by cardiac puncture into two volumes of distilled water and the brain removed. After suitable sample preparation, the concentration of test compound in blood and brain was determined by UPLC–MS/MS using a Quattro Premier XE (Waters, USA). For each mouse at each time point, the concentration in brain (ng g^−1^) was divided by the concentration in blood (ng mL^−1^) to give a brain/blood ratio. The mean value obtained was quoted.

*Ethics*. All regulated procedures on living animals were carried out under the authority of a project license issued by the Home Office under the Animals (Scientific Procedures) Act 1986, as amended in 2012 (and in compliance with EU Directive EU/2010/63). License applications will have been approved by the University's Ethical Review Committee (ERC) before submission to the Home Office. The ERC has a general remit to develop and oversee policy on all aspects of the use of animals on University premises and is a subcommittee of the University Court, its highest governing body.

*Parasite and NMT assays*: These were carried out as described previously.[8–10]

## Supporting Information

As a service to our authors and readers, this journal provides supporting information supplied by the authors. Such materials are peer reviewed and may be re-organized for online delivery, but are not copy-edited or typeset. Technical support issues arising from supporting information (other than missing files) should be addressed to the authors.
