# Supplementary material for: Development of Small-Molecule Trypanosoma brucei N-Myristoyltransferase Inhibitors: Discovery and Optimisation of a Novel Binding Mode
Source: ChemMedChem. 2015 Sep 23;10(11):1821–36. doi: 10.1002/cmdc.201500301 (PMC4648043; doi:10.1002/cmdc.201500301)
Supplement: Supplementary file 1 — Supplementary [file cmdc0010-1821-sd1.pdf]

CHEMMEDCHEM
Chemistry & Drug Discovery

Supporting Information

Development of Small-Molecule *Trypanosoma brucei* *N*-Myristoyltransferase Inhibitors: Discovery and Optimisation of a Novel Binding Mode

Daniel Spinks, Victoria Smith, Stephen Thompson, David A. Robinson, Torsten Luksch, Alasdair Smith, Leah S. Torrie, Stuart McElroy, Laste Stojanovski, Suzanne Norval, Iain T. Collie, Irene Hallyburton, Bhavya Rao, Stephen Brand, Ruth Brenk, Julie A. Frearson, Kevin D. Read, Paul G. Wyatt, and Ian H. Gilbert*[a]

cmdc_201500301_sm_miscellaneous_information.pdf

Table. Data measurement and model refinement statistics for *Lm*NMT protein:ligand complexes described in this work.

	LmNMT:**6**	LmNMT:**7**	LmNMT:**13**	LmNMT:**14**	LmNMT:**44**
PDB code	5AG5	5AG4	5AG6	5AG7	5AGE
**Data Measurement**					
Source	ESRF ID14eh1	DLS I03	Micromax 007	ESRF ID14eh1	Micromax 007
Space Group	$P2_1$	$P2_1$	$P2_1$	$P2_1$	$P2_1$
Unit Cell Dimensions (Å)	a=48.5, b=90.8, c=53.3 β=114.1	a=48.7, b=90.9, c=53.9 β=113.5	a=48.7, b=90.3, c=53.3 β=114.6	a=48.2, b=91.2, c=53.2 β=113.7	a=48.0, b=90.2, c=53.0 β=113.8
Resolution (Å)[a]	50.0-2.00 (2.07-2.00)	20.0-2.00 (2.07-2.00)	44.0-2.0 (2.10-2.00)	20.0-2.60 (2.69-2.60)	20.0-2.0 (2.03-2.00)
Observations	77611	94047	84697	45867	54477
Unique Observations	28183	28546	26648	13076	25800
Rmerge (%)[a]	3.6 (12.1)	4.5 (40.3)	7.7 (32.3)	7.5 (44.5)	5.7 (35.6)
I/σI[a]	33.2 (11.9)	23.5 (1.7)	11.4 (3.5)	11.6 (1.9)	21.8 (2.7)
Completeness (%)[a]	98.5 (98.6)	97.8 (84.3)	93.4 (87.8)	99.9 (99.9)	92.3 (85.6)
Redundancy[a]	2.8 (2.7)	3.3 (2.7)	3.2 (3.1)	3.5 (3.5)	2.1 (1.9)
**Refinement Statistics**					
Resolution Range (Å)	48.7 – 2.00	20.0-2.00	40.0-2.00	20.0-2.60	19.7 – 2.00
R-factor (R_{work}/R_{free})	16.5/21.6	18.2/24.6	17.3/23.6	17.4/26.7	17.9/24.1
Number of atoms[b]	3345/63/22/275	3345/63/38/125	3269/63/22/300	3345/63/17/95	3345/63/29/185
Mean B-factor ($Å^2$)[b]	28/17/22/32	40/31/39/44	19/14/19/28	36/29/47/35	31/25/34/37

RMS bond length deviation (Å)	0.024	0.024	0.023	0.016	0.023
RMS bond angle deviation (°)	1.981	2.04	1.99	1.72	2.01

.[a] Data for highest resolution shell in partenthesis. [b] Number of atoms and mean B-factors for non-hydrogen atoms from protein, co-factor, ligand and solvent respectively.
